# Developing SHAP interpretable machine learning models for assessing biopsychosocial risk in female drug users: a small sample study

**DOI:** 10.3389/fpsyt.2026.1736274

**Published:** 2026-04-22

**Authors:** Xiao Wang, Mingjian Gao, Jialin Gao, Fei Geng, Xiqing Yuan, Xinyu Cheng, Qiulian Xing, Yu Ming, Gancheng Zhu, Huimin Shi, Peng Wang

**Affiliations:** 1 School of Psychology, Nanjing Normal University, Nanjing, China; 2 Faculty of Health and Wellness, City University of Macau, Macao, Macao SAR, China; 3 Shanghai Female Drug Rehabilitation Center, Shanghai, China; 4 School of Psychology, Shandong Normal University, Jinan, China; 5 School of Digital Creativity, Shandong College of Electronic Technology, Jinan, China; 6 Center for Psychological Sciences, Zhejiang University, Hangzhou, China; 7 School of Health Science, Shandong University of Traditional Chinese Medicine, Jinan, China; 8 School of Education, Wenzhou University, Wenzhou, China

**Keywords:** risk assessment, small sample, imbalanced data, oversampling, machine learning classification, SHAP interpretability analysis

## Abstract

**Background:**

While interventions for female drug users have received considerable attention, comprehensive and objective risk assessment tools—particularly those integrating biopsychosocial dimensions—remain lacking, despite their critical public health need.

**Objective:**

This study aimed to develop a comprehensive assessment model for evaluating the physiological, psychological, and social risks among female drug users. Methods: Based on the biopsychosocial model, variables of the five dimensions of physiological function, psychological and cognitive function, drug dependence, social support, and self-control were collected from 96 participants. Professionals rated these participants on the five dimensions and the total risk. These ratings and variables served as inputs and outputs for our classification model. We oversampled the data and evaluated the classification of 6 classifiers.

**Results:**

Firstly, the machine learning classification results for the 5-dimensional risk and total risk performed relatively well. Next, oversampling improved the classification performance for most dimensions (except social support risk) and the total risk assessment. After oversampling, the true strengths of different algorithms became more apparent, with Random Forest and Logistic regression emerging as the optimal classifier for multiple dimensions. Thirdly, by applying SHAP, a novel interpretability method, we identified key variables in each dimension and for the total risk, thereby enhancing the transparency of the model’s decisions.

**Conclusion:**

The machine learning model, encompassing physiological, psychological, cognitive, drug refusal, social support, and self-control dimensions, modestly but effectively identified at-risk populations of female drug users. It should be noted that the current study is limited by the sample size of 96 participants; to address this, we plan to expand the sample in future research to overcome this constraint and reduce potential model overfitting. The developed model holds promise for researchers seeking to pinpoint at-risk female drug abusers, facilitating targeted interventions and corrections.

## 1 Introduction

Among the drug-using population, female drug users experience greater social stigma and psychological pressure, exemplified by a lack of gender-specific professional support, economic hardships, and heightened discrimination due to gender (1, 2). These compounded stressors contribute significantly to increased susceptibility to depression, anxiety, and other emotional disturbances (3). Current research on drug users primarily focuses on two areas: first, interventions such as those combining exercise training and pharmacotherapy (4), integrated treatment merging behavioral and cognitive approaches (5), psychological and social interventions (6), and comprehensive methods incorporating pharmacological, psychological, and social support (7); second, risks associated with drug use, such as drug dependence (8), psychological disorders (9), and other related health complications(eg, infectious diseases (10)).

### 1.1 Model framework

Skewes and Gonzales (11, 12) proposed a biopsychosocial model of addictive behavior based on Engel’s biopsychosocial model, which includes biological factors (such as genetics and physical condition), psychological and cognitive factors (such as depression and cognitive training), and social factors (such as peer relationships) (13). Guided by this biopsychosocial model, the present study aims to assess the total risk level faced by women following drug use. The assessment incorporates indicators across three dimensions: physiological, psychological and cognitive, and social support.

First, drug use leads to a decline in overall health and increases the risk of chronic diseases. Studies have reported a high prevalence of chronic conditions among individuals hospitalized due to drug use (14), such as vascular diseases (15). Furthermore, improving physical health through exercise interventions is a core objective in the treatment of individuals with drug addiction (16). In this study, physiological functional capacity and the prevalence of chronic diseases among female drug users are included in the assessment.

Secondly, drug use can lead to changes in an individual’s mental health and cognitive function. Studies have reported a high prevalence of depressive symptoms among drug users (9), while impaired cognitive function (e.g., reduced attention) (17)and decreased self-efficacy (18)are also commonly observed. In assessing psychological and cognitive risks, this study incorporates general mental health variables (e.g., depression, anxiety) as well as cognitive performance tests (e.g., outcomes of cognitive training related to addiction). Additionally, impulsive behavior has been identified as a typical symptom following drug use (19). Self-control is treated as a distinct dimension within psychological risk, evaluated through indicators such as level of self-control and emotional stability to specifically assess impulse control risks in female drug users. Beyond general psychological risks, craving for drugs is a specific emotional state commonly experienced by drug users (20), referring to the intense desire to obtain and use drugs and the anticipated pleasure associated with such use. Accordingly, this study considers “drug dependency” as another separate dimension in psychological risk, incorporating indicators such as time to relapse and drug use tolerance (to measure craving intensity). Thirdly, drug use can alter an individual’s social support system. To gain social support and alleviate loneliness, drug users may form “drug-using social circles” (21, 22). Furthermore, due to social stigma and physical impairment, they often face difficulties in securing employment to sustain their livelihoods, frequently ending up in low-wage and unstable jobs (23). As societal acceptance of this population is generally limited, their overall level of social support tends to be low (24). Therefore, when assessing social support risks, this study incorporates indicators such as vocational skills acquired in institutions, level of social security (e.g., medical insurance and educational background), and social interaction patterns, to systematically evaluate the social support risks faced by female drug users.

When assessing risks among female drug users, professionals consider not only independent dimensions—such as the physiological, psychological, and social aspects—but also conduct a comprehensive evaluation of their total risk level. Therefore, this study integrates indicators across all dimensions as predictors to systematically assess the total risk faced by female drug users. Previous studies on risk assessment of female drug users have been limited, and most of them have focused on a single dimension, such as psychological or social. Existing studies mainly rely on self-report scales, which are susceptible to social approval biases and are difficult to avoid the tendency to cover up in responses. To this end, this study integrates multidimensional objective indicators, including scale assessment, physiological indicators, standardized cognitive tasks, and behavioral observation, to construct a comprehensive risk assessment framework. Compared with evaluation tools that rely on a single source or strong subjectivity, the use of multimodal data fusion can effectively reduce self-report bias and common method bias, thereby improving the prediction validity of the model and enhancing the ecological validity of the evaluation results.

### 1.2 Machine learning

Machine learning classifiers perform well in mining data from risk assessment and medical fields, like identifying individuals at risk of suicide (25), identifying individuals with heart disease (26), and identifying individuals in need of care (27), etc. Researchers have also conducted many investigations on the topic of substance use based on machine learning classification: researchers have determined users’ social media use disorders based on their usage traces in social media and found that machine learning can distinguish individuals with social media use disorders (28), and found that common classifiers based on individuals’ socioeconomic and lifestyle information was found to be effective in determining the smoking and drinking habits (29); one researcher analyzed resting-state functional imaging data of methamphetamine-dependent and general individuals and found that a machine learning classifier was able to distinguish images of the two groups (30).

In machine learning classification tasks, researchers often need to handle class-imbalanced data, which refers to an uneven distribution of samples across different categories (31). If an imbalanced dataset is used directly for training, it is likely to lead to biased classification results. To address this issue, resampling techniques such as oversampling are widely adopted. For example, the SMOTE method generates synthetic minority class samples through linear interpolation, thereby enhancing their representation and mitigating the model’s tendency to overfit due to data imbalance (32). Empirical studies have demonstrated that the application of oversampling techniques improves classifier performance in tasks such as rockburst prediction (33), software defect detection (34), and diabetes data analysis (35).

Shapley Additive Explanation (SHAP), based on Shapley values, quantifies the contribution of each feature to risk classification outcomes in machine learning classification models (36, 37). This method has been applied in various clinical scenarios, such as supporting clinical prediction and visualization of Alzheimer’s disease (38), analyzing psychosocial factors in adolescent substance use behaviors (38), and screening for low-cost biomarkers of attention deficit hyperactivity disorder (39). These studies collectively demonstrate that SHAP can effectively bridge machine learning models with clinical decision-making, enhancing model transparency and practical utility. To further improve the clinical interpretability of the risk assessment model for female drug users, this study employs the SHAP method to visually analyze and interpret key risk indicators within the model.

This study employs a machine learning model to construct a risk assessment framework for female drug users, primarily based on the following three advantages. First, machine learning can effectively integrate multimodal data without imposing strict assumptions on variable distributions or linear relationships. Second, it is capable of identifying complex nonlinear interactions among risk factors. Third, the model incorporates the SHAP interpretability analysis method, which can clearly reveal the key indicators driving risk classification and their respective contributions. Therefore, the combination of the machine learning model and the SHAP method adopted in this study achieves a balance between predictive performance and model interpretability, providing a reliable foundation for subsequent clinical intervention and applied research.

### 1.3 Current study

Building upon the biopsychosocial framework and existing medication evaluation systems (40), this study employed machine learning classification to assess the risk levels of female drug users across five dimensions (physiological function, psychological and cognitive function, drug dependency, social support, and self-control) as well as their total risk. To enhance model interpretability and identify key predictive variables within each dimension, the SHAP technique is applied for attribution analysis.

The following hypotheses were formulated:

Hypothesis 1: The study expected that classifiers could predict the risks of female drug users.

Hypothesis 2: The study expected that oversampling could improve the risk assessment results of female drug users.

## 2 Materials and methods

### 2.1 Participants

We recruited 96 participants in Shanghai, based on the principle of voluntary participation. The study was conducted at one of the few local rehabilitation centers that admit and provide long-term care for women who use drugs, offering a relatively accessible and well-defined population for research. We collected 66 variables from 96 female drug users, and five aspects were covered: physiological function, psychological and cognitive functions, drug dependence, social support, and self-control. The participants’ demographics were shown in **Table 1**.

**Table 1:** Demographics of female drug users (N = 96).

Category	Subcategory	Frequency
age	20–30 years old	10
	30–40 years old	26
	40–50 years old	38
	50–60 years old	17
	60–70 years old	5
duration of drug use	2 years	6
	2–5 years	18
	5–10 years	23
	More than 10 years	49
types of drug use	Opioids	9
	Psychedelics, Stimulants	87
education	Elementary school and below	16
	Junior high school/vocational school/technical school/junior college	61
	High school/vocational high school/college	16
	College and above	3

Due to the need to protect the privacy of the participants, the datasets generated and analyzed during this study are not publicly available but can be obtained from the corresponding author upon reasonable request. This study was carried out in accordance with the recommendations of the World Medical Association’s Declaration of Helsinki. The protocol was approved by the Ethics Committee of Shandong Normal University (No. sdnu-2021-08-16-01).

Expert judgment is a widely accepted approach in clinical risk assessment. Professionals with extensive experience in female drug user rehabilitation conducted multidimensional risk assessments covering physiological function, psychological and cognitive function, drug dependence, social support, self-control, and total risk. Prior to the assessment, all raters received targeted training to ensure a consistent understanding of the rating criteria across all dimensions. These expert evaluations are considered clinically valid, as the raters possessed specialized knowledge and maintained longitudinal engagement with the participants, enabling them to base their judgments on comprehensive behavioral and clinical information. The professional ratings reflect an overall assessment of risk severity across multiple domains. To identify individuals with the highest level of risk—those most in need of intensive intervention—we classified participants scoring above the 75th percentile as “high-risk” (label = 1), consistent with prior risk-stratification studies in clinical and behavioral health contexts (41, 42). Those at or below the 75th percentile were labeled as “low-risk” (label = 0).

### 2.2 Measures

#### 2.2.1 Questionnaire

General Information Questionnaire. A questionnaire was administered to collect information on the age, education level, marital status, and years of drug use.

Self-Rating Depression Scale (SDS). The scale consists of 20 items and is scored on a four-point scale. The total score of the 20 items is calculated and multiplied by 1.25 to obtain the standard score. A standard score of less than 50 indicates no depression, 50–60 indicates mild depression, 60–70 indicates moderate to severe depression, and 70–80 indicates severe depression. This scale has an α coefficient of 0.78 when measuring a drug-using population (43).

Self-Rating Anxiety Scale (SAS). The scale consists of 20 items and is scored on a four-point scale. The total score of the 20 items is calculated and multiplied by 1.25 to obtain the standard score. A standard score of less than 50 is no anxiety, 50–59 is mild anxiety, and more than 60 is severe anxiety. This scale has an α coefficient of 0.76 when measuring a drug-using population (43).

Drug Craving Scale. The scale, developed by Luo, consists of 34 items scored on a 7-point scale (22). The total score of 34 items is calculated, and the higher the score is, the higher the drug craving level. This scale has an α coefficient of 0.90 when measuring a drug-using population (44).

Chinese Self-Evaluations Scale (CSES). The scale, developed by Judge and adapted from Chinese by Du et al., contains 10 items and is scored using a 5-point Likert scale (45). The scale contains both positive and negative evaluations of the individual. The alpha coefficient for this scale is 0.83 (45).

Positive Psychology Capital. The scale, developed by Zhang et al., consists of 26 items and is scored using a 7-point Likert scale. The total score is calculated, and the higher the score is, the higher the level of psychological capital of the individual. The alpha coefficient for this scale is 0.90 (46). The questionnaire was used to measure female drug users’ self-efficacy and self-regulation levels.

Symptom Checklist 90 (SCL-90). The scale has 90 questions, which evaluates the mental health of the participants from 10 dimensions such as feeling, emotion, thinking, consciousness, and behavior. The scale score is scored on a 5-point scale, and the higher the total score of SCL-90, the lower the level of mental health (47).

16 Personality Factor(16PF). This study utilized Cattell’s Sixteen Personality Factor Questionnaire (16PF) to assess fundamental personality traits (48). The scale has 187 items. The questionnaire is designed to measure sixteen relatively independent primary personality factors, which are: Warmth (A), Reasoning (B), Emotional Stability (C), Dominance (E), Liveliness (F), Rule-Consciousness (G), Social Boldness (H), Sensitivity (I), Vigilance (L), Abstractedness (M), Privateness (N), Apprehension (O), Openness to Change (Q1), Self-Reliance (Q2), Perfectionism (Q3), and Tension (Q4). The psychological health score—a composite index reflecting emotional stability and adaptive functioning—was used in this study. To facilitate interpretation, this continuous score was divided into five ordinal levels (1 to 5) based on sample quintiles, with higher levels indicating lower psychological health.

#### 2.2.2 Other indicators

This study employed Computerized Cognitive Addiction Training (CCAT) (49) and an Equipment Performance (EP) (40)test to systematically assess risk dimensions in psychological and cognitive functioning among female drug users, to identify potential deficits in cognitive abilities.

CCAT is designed to mitigate core cognitive impairments associated with addiction through targeted training. It covers five cognitive domains: (1) Spatial Memory (Memory Blocks), (2) Working Memory (Object Guessing), (3) Cognitive Bias Modification (Color Matching), (4) Reasoning Ability (Raindrop Calculation), and (5) Attention (Focused Attention), to enhance executive control and reduce cognitive bias and impulsive behavior. Performance across these five tasks is evaluated using metrics including accuracy rate, score, mean correct response time, and mean incorrect response time.

The EP test was used to assess participants’ sensorimotor coordination, incorporating the “Intelligent Finger Dexterity Apparatus” (evaluating finger dexterity and hand-eye coordination through plugging, flipping, and rotating actions) and the “Intelligent Bimanual Coordination Apparatus” (measuring bimanual coordination and attention allocation). Test scores serve as the primary evaluation metric.

### 2.3 Procedure

#### 2.3.1 Software

Microsoft Excel was used to organize and store data. Then, data analysis was performed using PyCharm 2023.3.2, including NumPy, scikit-learn.

#### 2.3.2 Data collection

This study employed cluster sampling to recruit 96 female drug users, achieving a 100% questionnaire response rate. Data collection involved two phases: demographic surveys followed by psychological and behavioral assessments. To minimize testing fatigue, the CCAT, EP, and drug refusal ability tests were administered over three separate days. All participants provided written informed consent before participation, with explicit acknowledgement of their right to withdraw. Written statements were provided before each assessment, confirming that data would be used solely for scientific research with guaranteed confidentiality of all personal information.

#### 2.3.3 Machine learning classifiers

The study selected six representative classifiers that span major algorithmic families and balance predictive performance with interpretability—key considerations for clinical risk modeling. Decision Trees (DT) provide transparent, rule-based predictions by splitting data based on entropy (50). Random Forest (RF) and Extreme Gradient Boosting (XGBoost) extend tree-based approaches through ensemble learning: RF aggregates many randomly built trees to reduce overfitting (51), while XGBoost sequentially corrects prediction errors to achieve high accuracy (52). For linear and probabilistic baselines, we included Logistic Regression (LR), which maps features to class probabilities via a logistic function (53), and Naive Bayes (NB), which applies Bayes’ theorem under a feature independence assumption (54). Finally, Support Vector Machine (SVM) was added as a margin-based method that maximizes separation between classes (55). Together, these models reflect diverse strategies commonly used in behavioral and clinical prediction tasks, enabling a robust comparison of both performance and practical interpretability.

#### 2.3.4 Data pre-processing

To retain all available participants and maintain analytical consistency, missing entries were imputed using mean substitution, i.e., each missing value was replaced with the mean of the observed values for that variable across the entire sample. The data preprocessing was implemented in three sequential steps. Initially, the data were normalized to scale all feature attributes to the (0, 1) interval, ensuring uniform feature magnitudes for optimal algorithm performance (56). Subsequently, a 5-fold cross-validation scheme was applied, partitioning the dataset into five subsets and iteratively using four for training and one for validation, with final performance obtained by averaging all cycles to maximize data utility and prevent overfitting (57). Finally, a comprehensive grid search was conducted, systematically traversing predefined parameter spaces (detailed in **Table 2**) to identify the optimal parameter combinations for each classifier.

**Table 2:** Parameter settings for six classifiers.

Classifiers	Hyperparameters
DT	max_depth: [None, 5, 10, 20]; min_samples_split: [2, 5, 10]
RF	n_estimators: [50, 100, 200]; max_depth: [None, 10, 20]; min_samples_split: [2, 5, 10]
SVM	C: [0.01, 0.1, 1, 10]; kernel: [‘linear’, ‘rbf’]; gamma: [‘scale’, ‘auto’]
LR	C: [0.01, 0.1, 1, 10]; penalty: [‘l1’, ‘l2’]
XGBoost	n_estimators: [50, 100, 200]; max_depth: [3, 6, 9]; learning_rate: [0.01, 0.1, 0.3]
NB	None

The dataset exhibited a class imbalance with a 23:73 ratio between high-risk (Label=1) and low-risk (Label=0) participants. To address this, the SMOTE oversampling technique was applied to synthesize new minority-class instances, thereby balancing the category distribution for improved classification assessment. Importantly, SMOTE was implemented strictly within the cross-validation loop: for each fold, it was applied only to the training subset after the train-validation split, ensuring that no synthetic samples influenced the validation set and preventing data leakage.

#### 2.3.5 Evaluation indicators

This study employed four key metrics—accuracy, precision, recall, and F1-score—to evaluate model performance, all widely recognized in classification tasks (58, 59). Accuracy measures the overall proportion of correctly classified instances, calculated as (TP+TN)/(TP+FN+FP+TN). Precision reflects the proportion of true positives among instances predicted as positive, expressed as TP/(TP+FP), while recall (True Positive Rate, TPR) indicates the proportion of actual positives correctly identified, given by TP/(TP+FN). The F1-score represents the harmonic mean of precision and recall, computed as 2×Precision×Recall/(Precision+Recall). The Receiver Operating Characteristic (ROC) curve visualizes the trade-off between TPR and False Positive Rate (FPR) across thresholds, with the Area Under the Curve (AUC) quantifying overall classifier performance (58). Based on these metrics, AUC served as the primary criterion for model selection across dimensions and total risk assessment, while recall was particularly emphasized to evaluate oversampling effectiveness given the study’s focus on identifying at-risk individuals (Label=1).

#### 2.3.6 Shapley additive explanation

The Shapley Additive Explanation (SHAP) framework was employed to interpret feature contributions to risk prediction among female drug users. Based on Shapley values from cooperative game theory (37), SHAP quantifies each feature’s marginal contribution to model predictions through the additive formula:


$$y_{i}\;=\;y_{base}+f\left(x_{i1}\right)+f\left(x_{i2}\right)\;+\ldots +\;f\left(x_{ij}\right)$$

Where $\;y_{i}$ is the predicted sample of i, $y_{base}$ is the baseline prediction, and $f\left(x_{ij}\right)$ represents the contribution of feature j.

A positive SHAP value indicates a feature increases predicted risk, while a negative value decreases it. This study used SHAP to visualize and analyze the top 20% most influential features for each risk dimension and the total risk.

**Figure 1:**
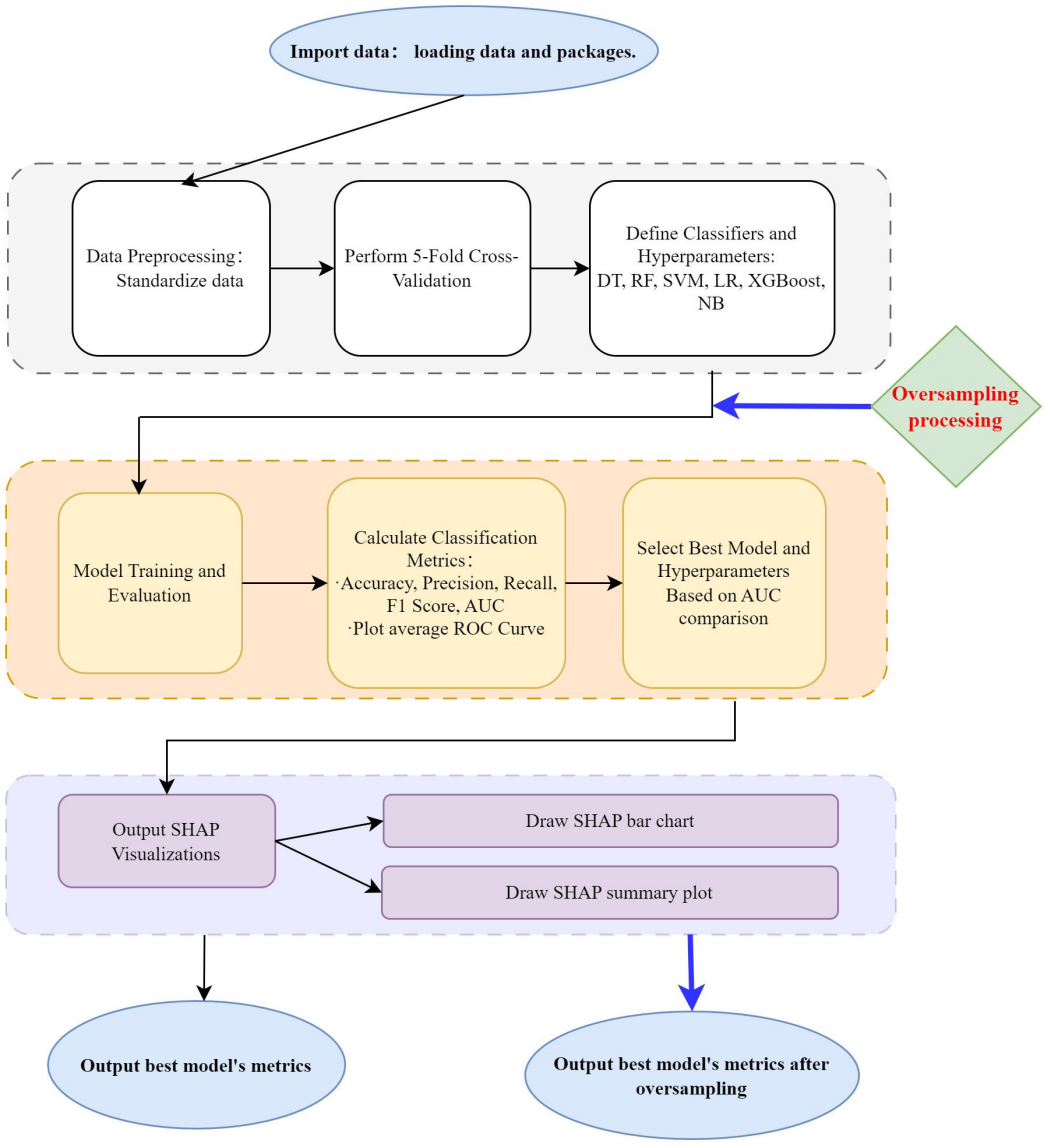
Machine learning classification and result output.

The machine learning classification process and SHAP visualization were shown in **Figure 1**. The code used in the study can be found in Appendix A.

## 3 Results

### 3.1 Results before oversampling

As summarized in **Table 3** and **Supplementary Table 1** (in Appendix B), all optimal models demonstrated satisfactory to good performance across classification metrics, outperforming random chance levels and thereby validating Hypothesis 1. For a concise presentation of the results, see the specific results of the 5 dimensions in Appendix B.

**Table 3:** Classifier indicator results.

Sampling condition	Classifier	Accuracy	Total precision rate	Total recall rate	Total F1 value	AUC
The total risk before oversampling	DT	0.64	0.51	0.52	0.49	0.54
	RF	0.71	0.67	0.49	0.45	0.62
	SVM	0.73	0.62	0.52	0.49	0.70
	LR	0.72	0.65	0.65	0.64	0.75
	XGBoost	0.72	0.66	0.57	0.55	0.60
	NB	0.47	0.56	0.58	0.46	0.61
The total risk after oversampling	DT	0.68	0.58	0.60	0.58	0.58
	RF	0.71	0.60	0.53	0.53	0.61
	SVM	0.69	0.60	0.56	0.51	0.59
	LR	0.68	0.62	0.64	0.61	0.74
	XGBoost	0.70	0.58	0.61	0.56	0.62
	NB	0.49	0.53	0.53	0.46	0.56

**Figure 2:**
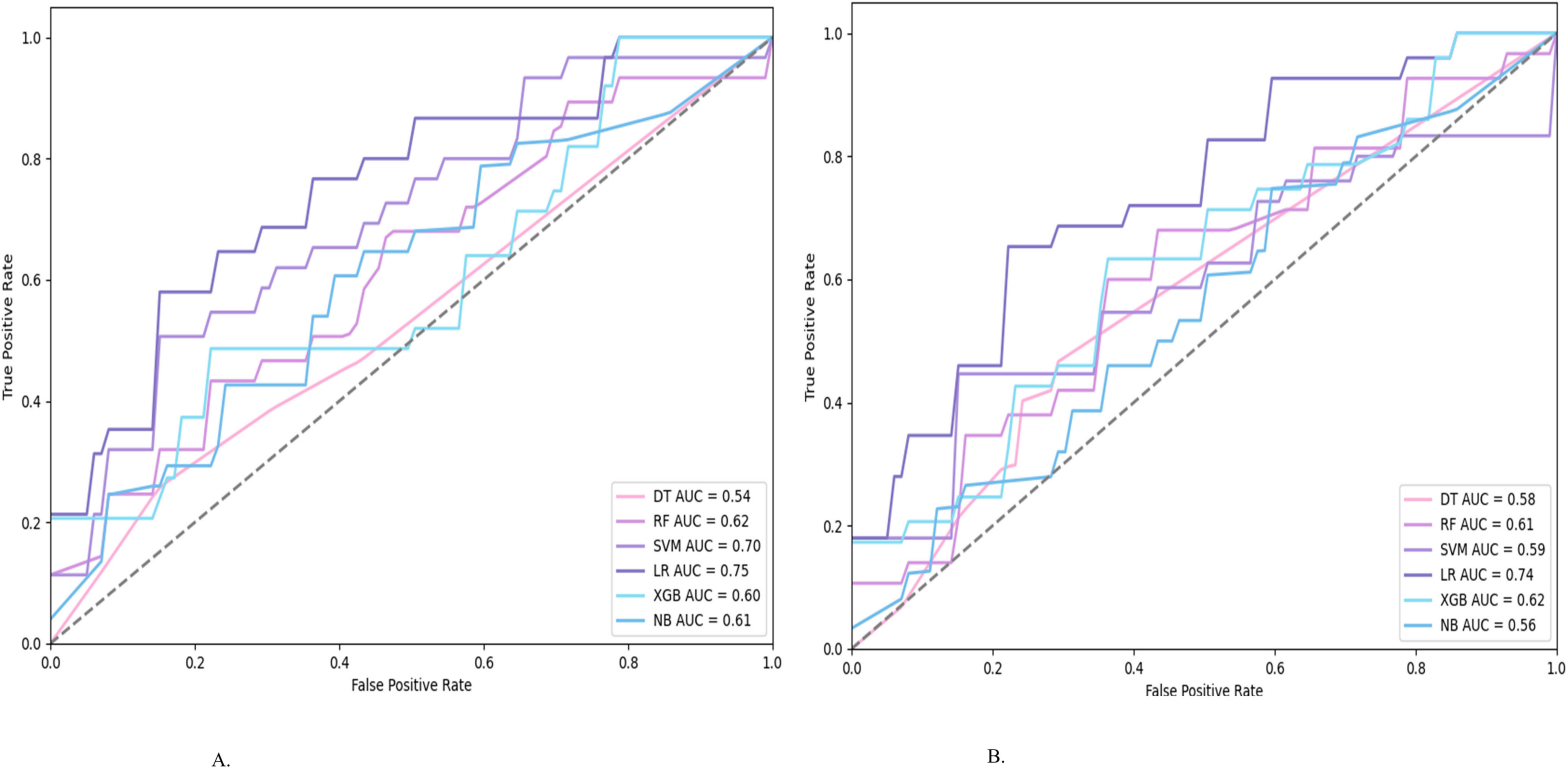
Total risk ROC curves. **(A)** ROC plot of the total risk before oversampling. **(B)** ROC plot of the total risk after oversampling.

Classification Metrics. When predicting the total risk, the accuracy ranged from 0.47 and 0.73, the total precision ranged from 0.51 and 0.66, the total recall ranged from 0.49 and 0.65, the total F1 score ranged from 0.45 and 0.64, and the AUC ranged from 0.54 and 0.75 (see **Table 3**). The ROC curves for all classifiers were shown in **Figure 2A**. Based on AUC as the primary selection criterion, logistic regression (LR) emerged as the best-performing model—likely due to its robustness in small-sample settings with moderate feature dimensionality, where complex models may overfit. All LR metrics fell between 0.64 and 0.75, indicating consistent and reliable performance.

**Figure 3:**
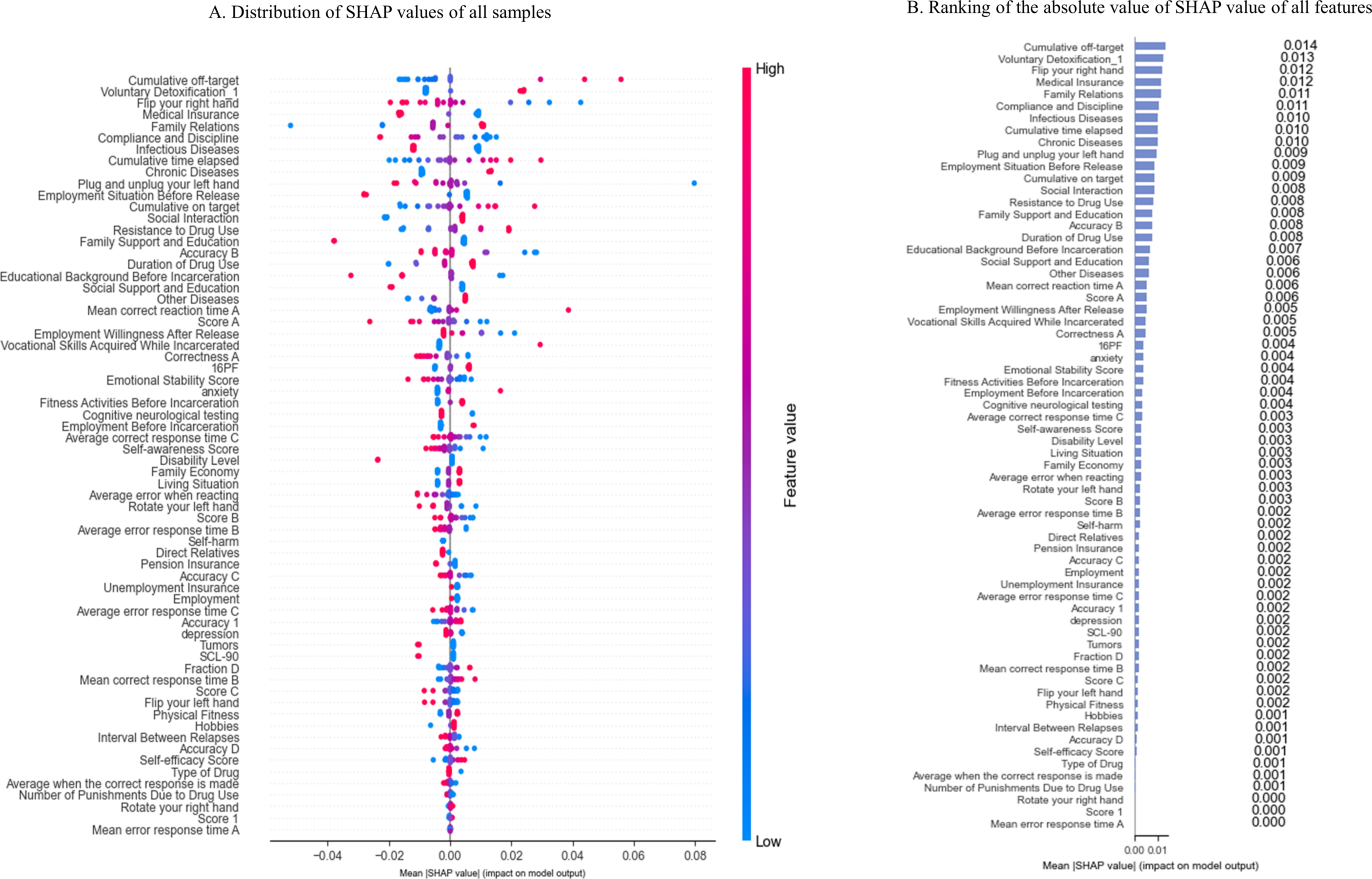
SHAP results for the total risk before oversampling. **(A)** Distribution of SHAP values of all samples before oversampling. **(B)** Ranking of the absolute value of SHAP value of all features after oversampling.

Feature Importance. **Figure 3A** displayed the SHAP values for the 66 predictors of total risk. Among the top 20% most influential features, the three with the largest absolute impact were “Cumulative off-target”, “Voluntary Detoxification_1”, and “Flip your right hand”. Notably, these features map onto core domains of neurocognitive and psychosocial functioning relevant to addiction recovery:

“Voluntary Detoxification_1” reflected intrinsic motivation—a well-established protective factor in substance use treatment;

“Flip your right hand”, a simple motor coordination task, likely served as a proxy for basic cognitive-motor integrity, which was frequently compromised in chronic drug users;

“Cumulative off-target” measured tracking errors across of EP task capturing attentional and bimanual coordination deficits linked to relapse risk.

Additional high-impact variables included “Medical Insurance”, “Family Relations”, “Compliance and Discipline”, “Infectious Diseases (reverse-scored) “, and “Employment Situation Before Release”. Critically, higher scores on these variables corresponded to negative SHAP values (red dots on the left in **Figure 3A**), indicating they function as protective factors: better access to healthcare, stronger family ties, institutional compliance, improved physical health, and pre-release employment stability were all associated with lower total risk.

Ranked by mean absolute SHAP value (**Figure 3B**), these 14 features dominated the model’s decision logic. They spanned all five predefined risk domains—5 related to psychological and cognitive function, 4 to social support, 2 to drug dependence, 2 to physiological function, and 1 to self-control—indicating that both psychosocial resources and neurocognitive functioning played a central role in shaping total risk profiles among female drug users in this sample.

### 3.2 Results after oversampling

According to the classification indicators in **Table 4**, the corresponding indicators of the optimal classification model for each dimension and total risk after oversampling were acceptable or good, which was better than the random level, and hypothesis 1 was verified. The dimensions and the total risk were presented in the following paragraphs. For a concise presentation of the results, see the specific results of the 5 dimensions in Appendix B.

**Table 4:** Average values of classification metrics before and after oversampling.

Dimension/Total Risk	Accuracy	Total precision rate	Total recall rate	Total F1 value	AUC
A	0.64	0.72	0.51	0.42	0.55
a	0.61	0.61	0.57	0.52	0.55
B	0.67	0.65	0.56	0.53	0.63
b	0.62	0.63	0.58	0.54	0.58
C	0.80	0.77	0.53	0.51	0.64
c	0.67	0.58	0.59	0.55	0.58
D	0.53	0.51	0.47	0.44	0.50
d	0.51	0.47	0.46	0.43	0.48
E	0.81	0.79	0.52	0.50	0.59
e	0.64	0.58	0.54	0.50	0.57
F	0.66	0.61	0.55	0.51	0.64
f	0.66	0.58	0.58	0.54	0.62

A to E represented dimensions 1 to 5, and F represents the total risk. The corresponding oversampled average classification indicators for all dimensions and total risk were a to f.

Classification Metrics. When predicting the total risk, the accuracy ranged from 0.49 and 0.71, the total precision ranged from 0.53 and 0.62, the total recall ranged from 0.53 and 0.64, the total F1 score ranged from 0.46 and 0.61, and the AUC ranged from 0.56 and 0.74(see **Table 4**). The ROC curves for all classifiers were shown in **Figure 2B**. LR again achieved the highest AUC and most balanced metrics (0.62–0.74), reinforcing its suitability for this imbalanced, small-sample context.

**Figure 4:**
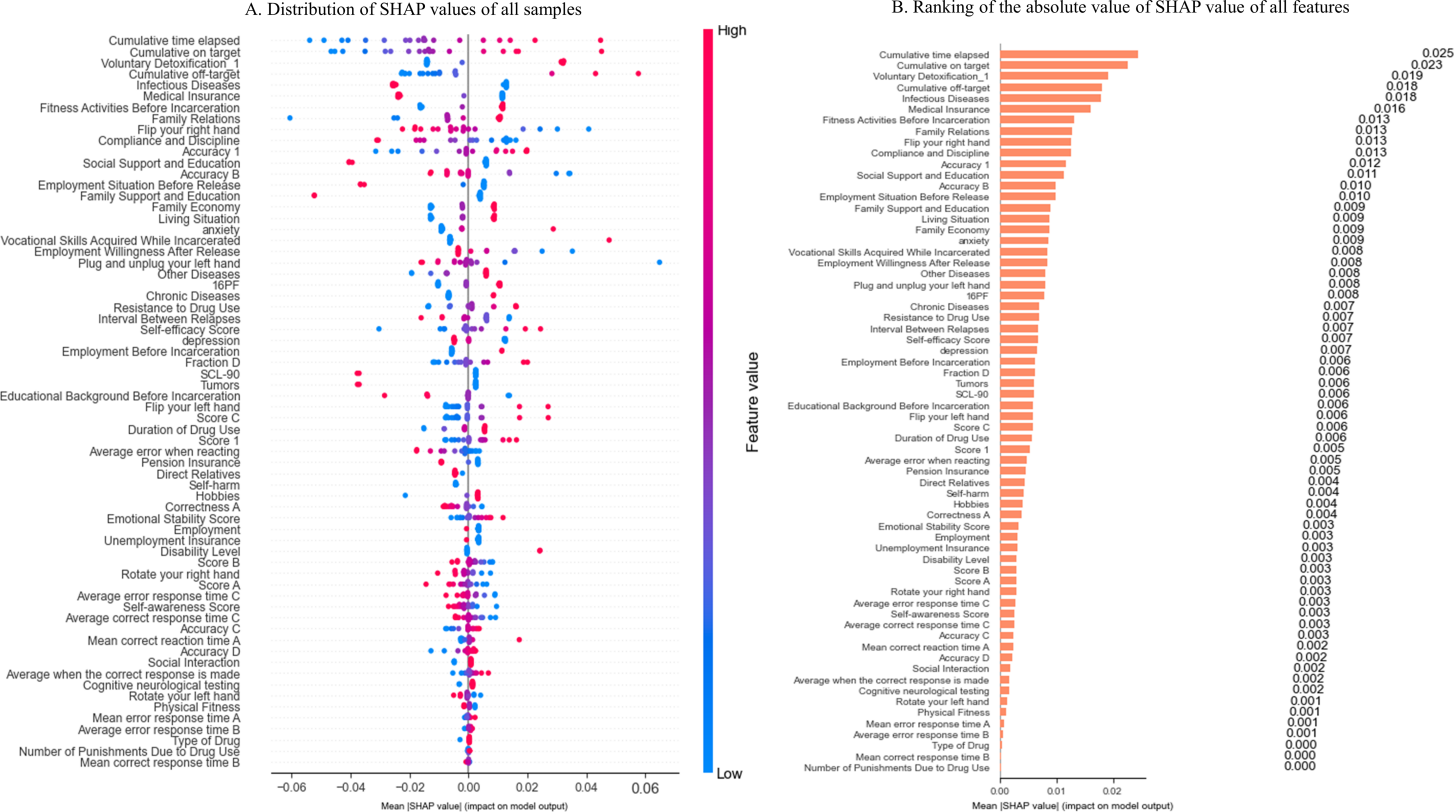
SHAP results for the total risk after oversampling. **(A)** Distribution of SHAP values of all samples before oversampling. **(B)** Ranking of the absolute value of SHAP value of all features after oversampling.

Feature Importance. **Figure 4A** displays the SHAP values for the 66 predictors of total risk after oversampling. Among the top 20% most influential features, the three with the largest absolute impact were “Cumulative time elapsed”, “Cumulative on target”, and “Voluntary Detoxification_1”. These features map onto key domains of treatment engagement and cognitive control:

“Cumulative time elapsed” captured the total time required to complete EP task, with longer durations potentially indicating slower cognitive processing or inefficient strategy adjustment;

“Cumulative on target”—the inverse of Cumulative off-target—measured sustained accuracy during the same EP task, reflecting stable attentional control and motor coordination;

“Voluntary Detoxification_1” again emerged as a robust protective indicator, consistent with its association with treatment motivation.

Eleven additional high-impact variables included “Cumulative off-target”, “Infectious Diseases (reverse-scored) “, “Medical Insurance”, “Fitness Activities Before Incarceration”, “Family Relations”, “Flip your right hand”, “Compliance and Discipline”, “Accuracy_1”, “Social Support and Education”, “Accuracy B”, and “Employment Situation Before Release”. Higher scores on 6 of these—”Infectious Diseases”, “Medical Insurance”, “Flip your right hand”, “Social Support and Education”, “Accuracy B”, and “Employment Situation Before Release”—corresponded to negative SHAP values (red dots on the left in **Figure 4A**), indicating they functioned as protective factors: better health status, social resources, pre-release stability, or cognitive performance were linked to lower total risk.

Ranked by mean absolute SHAP value (**Figure 4B**), these 14 features dominated the model’s decision logic. They spanned all five predefined risk domains—6 related to psychological and cognitive function, 5 to social support, and 1 each to drug dependence, physiological function, and self-control—underscoring that, even after oversampling, risk prediction remained anchored in neurocognitive and psychosocial factors, with minimal contribution from traditional substance-use or self-regulation indicators.

### 3.3 Comparison results of classification metrics before and after oversampling

Oversampling technology can improve the detection of potentially high-risk female drug users (**Table 4**). By comparing the average classification metrics before and after oversampling, it was found that the total recall of all dimensions and the total risk (except social support risk) increased, ranging from 0.02 to 0.06. This pattern supports Hypothesis 2, suggesting that addressing class imbalance enables the model to better identify true high-risk cases that were previously missed due to underrepresentation in the training data.

Prior to oversampling, optimal classifier selection was highly fragmented across risk domains, reflecting substantial instability in model performance under class imbalance. For instance, SVM yielded the highest AUC for drug dependence risk, while Random Forest (RF) performed best for physiological function, Naive Bayes (NB) for psychological and cognitive risk, XGBoost for drug dependence, Decision Tree (DT) for self-control risk, and Logistic Regression (LR) for total risk. This lack of coherence implies that, without balancing the sample, algorithmic performance was driven more by idiosyncratic data distributions than by robust, domain-general signal.

In contrast, after oversampling, classifier performance became markedly more consistent: Random Forest emerged as the dominant algorithm for three distinct domains—psychological and cognitive function, physiological function, and self-control risk—demonstrating its robustness in handling complex, imbalanced behavioral data. Logistic Regression achieved top performance for both drug dependence risk and total risk, likely benefiting from its calibration stability and interpretability in low-prevalence settings. XGBoost remained optimal only for social support risk, possibly due to the nonlinear interactions among socioeconomic indicators. Collectively, these results indicate that oversampling not only boosts sensitivity but also reveals more reliable and theoretically interpretable patterns of model superiority, reducing spurious variability in algorithm selection.

## 4 Discussion

### 4.1 The multidimensional risk structure faced by female drug users

This study reveals that the risks faced by female drug users form a complex system consisting of five relatively independent yet interconnected dimensions: physiological function, psychological and cognitive function, drug dependence, social support, and self-control. This structure is highly consistent with the biopsychosocial model of addiction, reflecting the multidimensional and complex nature of risks in this population. The results demonstrated that the optimal machine learning models developed in this study all performed better than chance level (AUC > 0.50). The classification performance ranged from acceptable to good, indicating that the classification models constructed for each dimension and the total risk are effective. The classification performance ranged from acceptable to modest across most classifiers, suggesting preliminary feasibility as a multidimensional risk screening tool.

The multidimensional risk assessment framework developed in this study integrates multi-source data, including scales, physiological functions, cognitive training results, and daily behavioral observations. The incorporation of diverse data types contributes to enhancing the ecological validity of the model. Furthermore, the framework can replace subjective evaluations traditionally conducted by professionals, as the classification outcomes generated by machine learning are more objective and quantifiable. In addition, as female drug rehabilitation participants progress through institutional treatment, the indicators are updated and reassessed over time, enabling the framework to support dynamic and real-time risk assessment, thereby significantly improving evaluation efficiency.

### 4.2 Key features of the total risks: interpretation based on SHAP

This study classified and predicted the total risk based on 66 indicators across five dimensions. The analysis revealed that indicators from all five dimensions contributed to the prediction of total risk. Among them, ten indicators consistently contributed to prediction both before and after oversampling, including: “Cumulative off-target (coordination ability score)”, “Voluntary Detoxification_1”, “Medical Insurance”, “Family Relations”, “Compliance and Discipline”, “Infectious Diseases (after reverse scoring)”, “Cumulative time elapsed (coordination ability score)”, “Flip your right hand (EP test - flexibility score)”, “Cumulative on target (coordination ability score)”, and “Employment Situation Before Release”. Among these, “Flip your right hand”, “Infectious Diseases (after reverse scoring)”, “Medical Insurance”, “Compliance and Discipline”, and “Employment Situation Before Release” were protective factors, meaning that higher scores on these indicators for female drug users were associated with lower total risk levels.

Based on the original data for total risk prediction, four additional indicators were unique and significant contributors: “Chronic Diseases”, “Plug and Unplug your left hand (coordination ability score)”, “Social Interaction”, and “Resistance to Drug Use (VR test score)”. After oversampling, the four unique contributing indicators were: “Fitness Activities Before Incarceration”, “Accuracy_1 (working memory ability score)”, “Social Support and Education”, and “Accuracy_B (attention training score)”. Among these, “Social Support”, “Education”, and “Accuracy_B (attention training score)” were protective factors.

According to the biopsychosocial framework, addictive behavior is both the outcome and the cause of the combined influence of biological, psychological, and social factors (2, 11). At the biological level, this study found that the degree of infectious diseases among female drug users positively contributed to the total risk. Existing surveys indicate that drug use promotes the occurrence of infectious diseases (60), which is consistent with the findings of this study. Therefore, when assessing the risk of female drug users, their infectious disease status should be given due attention.

In terms of psychological and cognitive function, attention should be paid to the attention, motor coordination, and flexibility demonstrated by female drug users in rehabilitation institutions. Better performance on various cognitive tests is generally associated with lower total risk levels. Drug use impairs cognitive functions such as attention (17). This study further indicates that attention and motor abilities (including flexibility and coordination) reflected in the CCAT test are executive function indicators that require close monitoring. Additionally, regarding drug craving—a unique emotional experience reflecting the degree of drug dependence—this study found that stronger voluntary detoxification motivation among female drug users was associated with higher total risk. This result may seem counterintuitive, but it is hypothesized that excessively high voluntary detoxification motivation may reflect intense psychological struggle, warranting caution regarding such high motivation states. Self-control is another important dimension in the psychological aspect. The study found that better daily behavioral performance among female drug users was associated with lower total risk. This aligns with existing research, which identifies self-control as a component of psychological capital that aids in the recovery of drug users (61). Daily behavioral performance serves as a quantifiable indicator of self-control. Therefore, when assessing the impact of self-control on total risk, close attention should be paid to its manifestation in daily behaviors.

At the social support, better family relationships, more comprehensive social security/welfare (such as medical insurance), and higher vocational skills acquired in rehabilitation institutions are associated with lower total risk levels among female drug users. Existing research has pointed out that jobs obtained by drug users after reintegrating into society are often low-paying and unstable (23), indicating significant challenges in social integration. Vocational skills acquired in institutions are closely related to post-release social integration, making them an important factor in social support risk assessment. Family, as another crucial source of social support, plays a key role in maintaining abstinence. Good family relationships help recovery (62), which is consistent with the findings of this study, suggesting that family relationships should be particularly emphasized when evaluating social support risks for female drug users. Furthermore, lack of social security/welfare (such as medical insurance) may hinder their rehabilitation process after returning to society (63), making it another important aspect of social support.

### 4.3 Oversampling technique: while it improves classification performance, application limitations remain

a notable feature of our results was the substantial instability in classifier performance across risk dimensions before oversampling. For instance, different algorithms emerged as optimal for different domains—SVM for social support risk, Naive Bayes for psychological/cognitive risk, XGBoost for drug dependence, and so on—with no single model consistently outperforming others. This fragmentation likely reflected three interrelated challenges: (1) limited sample size, which restricted reliable learning; (2) varying construct validity and measurability across risk domains (e.g., objective behavioral metrics versus subjective self-reports); and (3) differential sensitivity of machine learning algorithms to class imbalance and feature distribution. Under such conditions, it was unsurprising that model selection appeared erratic—particularly when predicting complex behavioral constructs that lacked a clear clinical gold standard. This instability underscored a critical point: in real-world rehabilitation settings, applying a uniform modeling approach across all risk domains may be neither feasible nor effective.

A comparison of classification performance before and after oversampling revealed that the oversampling technique improved the recall metrics across all dimensions (excluding the social support risk) and the total risk, indicating that it can enhance the classification performance on imbalanced small-sample datasets.

This study focuses on identifying high-risk groups among female drug users. Due to the category imbalance problem in the original data, the study employed oversampling techniques to increase the sample size of specific classes (i.e., high-risk categories). Similarly, in studies focusing on a few categories of predictions, such as predicting rockburst occurrence (33), and software defect detection (34), the use of oversampling technology has been found to improve classification indicators. In addition, it is found that the consistency of the classification model improves after oversampling, and RF and LR become the best classifiers, indicating that the classifier’s performance is related to the sample distribution in the original data. In conclusion, this study found that the use of oversampling technology in small sample imbalanced data has helped improve the classification index, explore the internal pattern of the data, and improve the consistency of model selection after determining the optimal classifier.

In addition, oversampling techniques have inherent limitations. In this study, classification metrics improved only modestly after oversampling (Δ = 0.02–0.06), suggesting a limited overall effect. Notably, recall and F1-score showed relatively greater gains, consistent with the goal of oversampling to enhance detection of the minority (high-risk) class. However, these comparisons are descriptive, based on point estimates from cross-validation. Given our modest sample size and the exploratory nature of the study, we did not perform formal statistical tests to assess the significance of these improvements. Accordingly, the observed gains should be interpreted as preliminary and hypothesis-supportive rather than definitive evidence of oversampling efficacy. We acknowledge this as a limitation and emphasize the need for future studies with larger, more diverse samples to rigorously evaluate resampling strategies in risk prediction for this population.

### 4.4 Limitations

The study is limited by its modest sample size (n = 96) and single-institution recruitment. Small samples increase the risk of model overfitting—particularly for complex algorithms—and may reduce the stability of feature importance rankings. Although we prioritized simpler, more robust models (e.g., logistic regression) and employed cross-validation and oversampling to mitigate these concerns, the findings should still be interpreted with caution. Generalizability is further constrained by the exclusive focus on female participants; future work should validate the model across diverse settings and populations, including male drug users and multi-site cohorts.

The data were processed using oversampling techniques, and while most classifier performance metrics showed descriptive improvements, the gains were modest for some models—and no formal statistical tests were conducted to assess the significance of these changes. Future research could rigorously evaluate the added value of oversampling in similar small, imbalanced clinical samples. In addition, there are many oversampling techniques available; given that this study focused on model construction, only SMOTE was employed. Subsequent work could compare multiple oversampling methods to identify optimal approaches.

It should be noted that risk labels were assigned by trained professionals based on their clinical judgment. However, due to practical constraints in the rehabilitation setting—such as variability in staff assignments and caseload fluctuations—participants were not consistently assessed by the same rater, and a multi-rater consensus protocol was not implemented. As a result, formal inter-rater reliability could not be evaluated. Although all raters received standardized training to enhance consistency in judgment, some degree of subjectivity may still remain.

### 4.5 Implications for practice, policy, and future research

Practice. Clinicians should routinely monitor cognitive functions—such as attention, coordination, and cognitive flexibility—as indicators of psychological functioning, while daily behavioral performance can serve as a quantifiable proxy for self-control. The counterintuitive positive association between motivation for voluntary detoxification and overall risk suggests that high levels of reported motivation should be interpreted with caution, as they may reflect ongoing psychological struggle rather than genuine readiness for change. At the biological level, infectious disease status should also be closely monitored, as it represents an important marker of elevated risk.

Policy. The protective roles of family relationships, medical insurance coverage, and vocational skills highlight the importance of investing in family-centered interventions, expanding access to healthcare, and providing skills training within rehabilitation facilities. Such efforts may facilitate successful social reintegration following release.

Future Research. Future studies should validate this model across more diverse populations and institutional settings. Moreover, while the present quantitative approach identified key patterns of risk, subsequent research could further enrich these findings by incorporating qualitative methodologies, such as Online Photovoice (OPV) (64), Online Interpretative Phenomenological Analysis (OIPA) (65), and Community-Based Participatory Research (CBPR) (66), to capture the lived experiences of female drug users and provide contextual insights that complement the risk patterns identified in this study.

## 5 Conclusion

The risk assessment of female drug abusers deserves attention. The machine learning model covering physiological, psychological and cognitive, drug dependence, social support, self-control, and total risk performed acceptably to well across most classifiers and successfully identified at-risk female drug users. The SHAP interpretability analysis revealed the impact of each variable on the prediction results in terms of 5 dimensions and total risk. It also identified the most influential variables (top 20%) in each dimension and total risk. The model can help researchers identify at-risk female drug abusers, which in turn can help to target interventions and corrections.

## Data Availability

The raw data supporting the conclusions of this article will be made available by the authors, without undue reservation.

## References

[1] Khazaee-Pool M Pashaei T Nouri R Taymoori P Ponnet K . Understanding the relapse process: exploring Iranian women’s substance use experiences. Subst Abuse Treatment Prevention Policy. (2019) 14:27. doi: 10.1186/s13011-019-0216-3, PMID: 31215472 PMC6582531

[2] Brown S . A whole-person approach to harm reduction for women. J Law Med Ethics. (2024) 52:45–51. doi: 10.1017/jme.2024.58, PMID: 38818596

[3] Li Z Ma Z Liu G Liu W Chen X . Research progress in localized group therapy for compulsory isolation drug rehabilitation. Chin Gen Pract. (2019) 22:128–35. doi: 10.3969/j.issn.1007-9572.2017.00.069

[4] Chen L Ru Q Xiong Q Yang J Xu G Wu Y . Potential Effects of Nrf2 in Exercise Intervention of Neurotoxicity Caused by Methamphetamine Oxidative Stress. In: Veskoukis AS , editor. Oxidative Medicine and Cellular Longevity. London: Hindawi vol. 2022 (2022). doi: 10.1155/2022/4445734, PMID: PMC9038420 35480870

[5] Haller M Norman SB Cummins K Trim RS Xu X Cui R . Integrated cognitive behavioral therapy versus cognitive processing therapy for adults with depression, substance use disorder, and trauma. J Subst Abuse Treat. (2016) 62:38–48. doi: 10.1016/j.jsat.2015.11.005, PMID: 26718130

[6] Fan X Zhang X Xu H Yang F Lau JTF Hao C . Effectiveness of a Psycho-Social Intervention Aimed at Reducing Attrition at Methadone Maintenance Treatment Clinics: A Propensity Score Matching Analysis. Int J Environ Res Public Health. (2019) 16:4337. doi: 10.3390/ijerph16224337, PMID: 31703302 PMC6888175

[7] Morgan N Daniels W Subramaney U . An inverse relationship between alcohol and heroin use in heroin users post detoxification. Subst Abuse Rehabil. (2020) 11:1–8. doi: 10.2147/sar.S228224, PMID: 32021548 PMC6955608

[8] Chen CY Storr CL Anthony JC . Early-onset drug use and risk for drug dependence problems. Addictive Behaviors. (2009) 34:319–22. doi: 10.1016/j.addbeh.2008.10.021, PMID: 19022584 PMC2677076

[9] Moulis L Le SM Hai VV Huong DT Minh KP Hai T . Gender, homelessness, hospitalization and methamphetamine use fuel depression among people who inject drugs: implications for innovative prevention and care strategies. Front Psychiatry. (2023) 14:1233844. doi: 10.3389/fpsyt.2023.1233844, PMID: 38025448 PMC10661402

[10] Lv J Jia Y Yan C Zhang X Xu K Xu J . Drug use behaviors and the risk of HIV infection among drug users in China between 2014 and 2021: cross-sectional study. JMIR Public Health Surveillance. (2024) 10:e56958–8. doi: 10.2196/56958, PMID: 39254571 PMC11407136

[11] Skewes MC Gonzalez VM . The biopsychosocial model of addiction. Principles Addict. (2013) 1:61–70. doi: 10.1016/B978-0-12-398336-7.00006-1, PMID: 38826717

[12] van den Ende MWJ Epskamp S Lees MH van der Maas HLJ Wiers RW Sloot PMA . A review of mathematical modeling of addiction regarding both (neuro-)psychological processes and the social contagion perspectives. Addictive Behaviors. (2022) 127:107201. doi: 10.1016/j.addbeh.2021.107201, PMID: 34959078

[13] Latt NNN Putdivarnichapong W Phetrasuwan S Vongsirimas N . Factors predicting the intention of drug abuse avoidance among adolescents in Pinlaung Township, Myanmar: predictive correlational design. BMC Public Health. (2024) 24:1–12. doi: 10.1186/s12889-023-17419-4, PMID: 38166869 PMC10759472

[14] Ingram I Deane FP Baker A Townsend CJ Collins CE Callister R . The health of people attending residential treatment for alcohol and other drug use: Prevalence of and risks for major lifestyle diseases. Drug Alcohol Rev. (2023) 42:1723–32. doi: 10.1111/dar.13752, PMID: 37715714

[15] Kwiatkowska W Knysz B Gąsiorowski J Witkiewicz W . Deep vein thrombosis of the lower limbs in intravenous drug users. Postępy Higieny i Medycyny Doświadczalnej. (2015) 69:510–20. doi: 10.5604/17322693.1150215, PMID: 25983290

[16] Guo J Zhang L Zhang L Li Y Yang S Sun Y . Effect of interactive exergame training on physical fitness and executive function among men with substance use disorder in rehabilitation center. Ment Health Phys Activity. (2024) 26:100598. doi: 10.1016/j.mhpa.2024.100598, PMID: 38826717

[17] Proebstl L Kamp F Koller G Soyka M . Cognitive deficits in methamphetamine users: how strong is the evidence? Pharmacopsychiatry. (2018) 51:243–50. doi: 10.1055/s-0043-123471, PMID: 29334687

[18] Maisto SA Connors GJ Zywiak WH . Alcohol treatment changes in coping skills, self-efficacy, and levels of alcohol use and related problems 1 year following treatment initiation. Psychol Addict Behav. (2000) 14:257–66. doi: 10.1037//0893-164x.14.3.257, PMID: 10998951

[19] Kozak K Lucatch AM Lowe DJE Balodis IM MacKillop J George TP . The neurobiology of impulsivity and substance use disorders: Implications for treatment. Ann New York Acad Sci. (2018) 1451:71–91. doi: 10.1111/nyas.13977, PMID: 30291624 PMC6450787

[20] Roozen HG van der Kroft P van Marle HJ Franken IHA . The impact of craving and impulsivity on aggression in detoxified cocaine-dependent patients. J Subst Abuse Treat. (2011) 40:414–8. doi: 10.1016/j.jsat.2010.12.003, PMID: 21315541

[21] Zhang SY . Control, peer association, and permissive attitudes to drug use: an integrated model explaining illicit drug use in China. Subst Use Misuse. (2021) 57:1–11. doi: 10.1080/10826084.2021.1991377, PMID: 34662263

[22] Luo Y . Research of the drug craving of those abstained from drugs during their treatment. Chongqing, China: Southwest Normal University (2004).

[23] McGinnis DM Ali SR . Evaluating SUD/OUD treatment outcomes related to vocational success for previously incarcerated persons: A review. IdeaExchange@UAkron. (2023) 5. Available online at: https://ideaexchange.uakron.edu/psychologyfromthemargins/vol5/iss1/5 (Accessed April 16, 2025).

[24] Nguyen H Dinh DX . Opioid relapse and its predictors among methadone maintenance patients: a multicenter, cross-sectional study in Vietnam. Harm Reduction J. (2023) 20:136. doi: 10.1186/s12954-023-00872-0, PMID: 37717002 PMC10505306

[25] Delgado-Gomez D Baca-Garcia E Aguado D Courtet P Lopez-Castroman J . Computerized Adaptive Test vs. decision trees: Development of a support decision system to identify suicidal behavior. J Affect Disord. (2016) 206:204–9. doi: 10.1016/j.jad.2016.07.032, PMID: 27475891

[26] Kumar Dubey A Choudhary K Sharma R . Predicting heart disease based on influential features with machine learning. Intelligent Automation Soft Computing. (2021) 30:929–43. doi: 10.32604/iasc.2021.018382

[27] Ioannidis K Serfontein J Deakin J Bruneau M Ciobanca A Holt L . Early warning systems in inpatient anorexia nervosa: A validation of the MARSIPAN -based modified early warning system. Eur Eating Disord Review. (2020) 28:551–8. doi: 10.1002/erv.2753, PMID: 32542781

[28] Marengo D Montag C Mignogna A Settanni M . Mining digital traces of facebook activity for the prediction of individual differences in tendencies toward social networks use disorder: A machine learning approach. Front Psychol. (2022) 13:830120. doi: 10.3389/fpsyg.2022.830120, PMID: 35350734 PMC8957912

[29] Abirami MS Vennila B Chilukalapalli EL Kuriyedath R . A classification model to predict onset of smoking and drinking habits based on socio-economic and sociocultural factors. J Ambient Intell Humanized Computing. (2020) 12:4171–9. doi: 10.1007/s12652-020-01796-4, PMID: 30311153

[30] Yu H Huang S Zhang X Huang Q Liu J Chen H . Identifying methamphetamine dependence using regional homogeneity in BOLD signals. Comput Math Methods Med. (2020) 2020:1–5. doi: 10.1155/2020/3267949, PMID: 41992061

[31] Cheng XF Li J Li XF . An imbalanced data classification algorithm based on under sampling. ComputercEngineering. (2011) 37:147–9. doi: 10.3969/j.issn.1000-3428.2011.13.047

[32] Lomax S Vadera S . A survey of cost-sensitive decision tree induction algorithms. ACM Computing Surveys. (2013) 45:1–35. doi: 10.1145/2431211.2431215

[33] Zhili T Xue W Qianjun X . Rockburst prediction based on oversampling and objective weighting method. J Tsinghua Univ (Science Technology). (2021) 61:543–55.

[34] Wang H Jiang F Du J Zhao J . Comparative study of oversampling and ensemble learning methods in software defect prediction. Comput Modernization. (2020) (6):83.

[35] Teh K Armitage P Tesfaye S Selvarajah D Wilkinson ID . Imbalanced learning: Improving classification of diabetic neuropathy from magnetic resonance imaging. Gwak J editor PloS One. (2020) 15:e0243907. doi: 10.1371/journal.pone.0243907, PMID: 33320890 PMC7737960

[36] Lundberg SA . Unified approach to interpreting model predictions. arXiv preprint arXiv:1705.07874. (2017):4768–77. Available online at: https://arxiv.org/abs/1705.07874 (Accessed April 16, 2025).

[37] Shapley L . Stochastic games. Proc Natl Acad Sci. (1953) 39:1095–100. doi: 10.1073/pnas.39.10.1953, PMID: 16589380 PMC1063912

[38] Govindarajan R Thirunadanasikamani K Napa KK Sathya S Murugan JS Priya KGC . Development of an explainable machine learning model for Alzheimer’s disease prediction using clinical and behavioural features. MethodsX. (2025) 15:103491. doi: 10.1016/j.mex.2025.103491, PMID: 40697328 PMC12281133

[39] Meng H Li S Xing X Fu R Li Y Liu Q . Machine learning–guided feature selection and predictive model construction for attention-deficit/hyperactivity disorder. Front Psychiatry. (2025) 16:1724359. doi: 10.3389/fpsyt.2025.1724359, PMID: 41480341 PMC12753890

[40] Wang DS Lv ZH Zu SQ . New exploration of education and treatment in a unified drug rehabilitation model: An example of “4 + 1+1 + 1” weekly education and treatment model in Shanghai Drug Rehabilitation Bureau. Crime Rehabil Res. (2020) (3):38–43.

[41] Dubow EF Huesmann LR Boxer P Smith C . Childhood and adolescent risk and protective factors for violence in adulthood. J Criminal Justice. (2016) 45:26–31. doi: 10.1016/j.jcrimjus.2016.02.005, PMID: 27524843 PMC4979576

[42] Jang Y . Feature-based ensemble modeling for addressing diabetes data imbalance using the SMOTE, RUS, and random forest methods: a prediction study. Ewha Med J. (2025) 48:e32. doi: 10.12771/emj.2025.00353, PMID: 40703379 PMC12277495

[43] Liang Y . An investigation on drug addiction features and mental health status of 232 compulsory abandoner of drug habits in dazhou city. J Leshan Normal Univ. (2017) 32:98–101.

[44] Zeng X Xiao X Dong S . Drug craving and relapsing tendency: The multiple mediating and regulatory effects. Chin J Clin Psychol. (2018) 26:947–51. doi: 10.16128/j.cnki.1005-3611.2018.05.024

[45] Du J Zhang X Zhao Y . Reliability, validation and construct confirmatory of core self-evaluations scale. psychol Res. (2012) 5:54–60.

[46] Luthans F Avolio BJ Avey JB Norman SM . Positive Psychological Capital: Measurement and relationship with performance and satisfaction. Personnel Psychol. (2007) 60:541–72. doi: 10.1111/j.1744-6570.2007.00083.x, PMID: 40046247

[47] Wang ZH Yu WL Shen Z Ye Y Hu L Yu GX . Reliability and validity of the symptom checklist 90 in Chinese professional females. Chin J Ind Med. (2017) 30:247–50. doi: 10.13631/j.cnki.zggyyx.2017.04.002

[48] Hao T Lingyu Z Danyang LI Encheng S Chuanhao MA . The personality analysis of synthetic drug addicts and relapse prevention strategies. Chin J Drug Depend. (2018) 27:58–63. doi: 10.13936/j.cnki.cjdd1992.2018.01.011

[49] Zhu Y Jiang H Su H Zhong N Li R Li X . A newly designed mobile-based computerized cognitive addiction therapy app for the improvement of cognition impairments and risk decision making in methamphetamine use disorder: randomized controlled trial. JMIR mHealth uHealth. (2018) 6:e10292. doi: 10.2196/10292, PMID: 29925497 PMC6031898

[50] Utgoff PE Berkman NC Clouse JA . Decision tree induction based on efficient tree restructuring. Mach Learn. (1997) 29:5–44. doi: 10.1023/A:1007413323501, PMID: 41886696

[51] Liaw A . Classification and regression by random Forest. R News. (2002) 2:18–22.

[52] Zounemat-Kermani M Batelaan O Fadaee M Hinkelmann R . Ensemble machine learning paradigms in hydrology: A review. J Hydrology. (2021) 598:126266. doi: 10.1016/j.jhydrol.2021.126266, PMID: 38826717

[53] Breiman L . Random forests. Mach Learn. (2001) 45:5–32. doi: 10.1023/A:1010933404324, PMID: 41886696

[54] Nguyen G Dlugolinsky S Bobák M Tran V López García Á Heredia I . Machine learning and deep learning frameworks and libraries for large-scale data mining: A survey. Artif Intell Review. (2019) 52:77–124. doi: 10.1007/s10462-018-09679-z, PMID: 30311153

[55] Hearst MA Dumais ST Osuna E Platt J Scholkopf B . Support vector machines. IEEE Intelligent Syst their Appl. (1998) 13:18–28. doi: 10.1109/5254.708428, PMID: 25079929

[56] Abadi M Barham P Chen J Chen Z Davis A Dean J . TensorFlow: a system for large-scale machine learning. 12th USENIX Symposium on Operating Systems Design and Implementation (OSDI '16). Savannah, GA: USENIX Association. (2016). 265–283.

[57] Hastie T Friedman J Tibshirani R . The elements of statistical learning: data mining, inference, and prediction. New York: Ny Springer New York Imprint: Springer (2009). doi: 10.1007/978-0-387-84858-7, PMID:

[58] Powers, D. M . Evaluation: from precision, recall and F-measure to ROC, informedness, markedness and correlation. J Mach Learn Technol. (2008) 2:37–63. doi: 10.9735/2229-3981

[59] Yıldız OT Aslan Ö Alpaydın E . Multivariate statistical tests for comparing classification algorithms. Coello CAC (ed.). Learning and Intelligent Optimization. LION 2011. Lecture Notes in Computer Science, 6683. Berlin: Springer. (2011). doi: 10.1007/978-3-642-25566-3_1, PMID:

[60] Zhang M He J Young JM You J Li J . Impact pathways of adverse childhood experiences on infectious diseases among substance abusers in border regions: structural equation modeling. Front Public Health. (2025) 13:1518607. doi: 10.3389/fpubh.2025.1518607, PMID: 40416651 PMC12098348

[61] Zhang SY Demant J . Effects of self-control, drug-use peers and family attachment on drug use among Chinese users: A gender-specific analysis. Drug Alcohol Rev. (2021) 40:1369–76. doi: 10.1111/dar.13295, PMID: 33858035

[62] Gek Hsia JT Yusoff NH . Pengaruh agen-agen kawalan sosial tidak formal dalam mewujudkan kesedaran dan sokongan untuk kekal pulih bekas pengguna dadah: Satu kajian literatur sistematik. Malaysian J Soc Space. (2024) 20:133–46. doi: 10.17576/geo-2024-2003-11

[63] Shaw A . Women in mid-life and older age in recovery from illicit drug use: connecting and belonging. Front Psychiatry. (2023) 14. doi: 10.3389/fpsyt.2023.1221500, PMID: 37636828 PMC10450501

[64] Tanhan A Strack RW . Online photovoice to explore and advocate for Muslim biopsychosocial spiritual wellbeing and issues: Ecological systems theory and ally development. Curr Psychol. (2020) 39:2010–25. doi: 10.1007/s12144-020-00692-6, PMID: 30311153

[65] Dari T Fox C Laux JM Speedlin Gonzalez S . The development and validation of the Community-Based Participatory Research Knowledge Self-Assessment Scale (CBPR-KSAS): A Rasch analysis. Measurement Eval Couns Dev. (2023) 56:64–79. doi: 10.1080/07481756.2022.2034478, PMID: 37339054

[66] Waalkes PL Mizutani Y Kim SR Napitupulu A Thompson J Leiva AN . Professional identity development of counselor educators with international backgrounds through online photovoice. Int J Adv Counsell. (2025) 47:150–70. doi: 10.1007/s10447-024-09582-z, PMID: 30311153

