# Supplementary material for: Developing SHAP interpretable machine learning models for assessing biopsychosocial risk in female drug users: a small sample study
Source: Front Psychiatry. 2026 Apr 22;17:1736274. doi: 10.3389/fpsyt.2026.1736274 (PMC13148034; doi:10.3389/fpsyt.2026.1736274)
Supplement: Supplementary file 1 [file SupplementaryFile1.docx]

import numpy as np
import pandas as pd
from sklearn.preprocessing import StandardScaler
from sklearn.model_selection import KFold, GridSearchCV
from sklearn.metrics import classification_report, roc_curve, auc
from sklearn.tree import DecisionTreeClassifier
from sklearn.ensemble import RandomForestClassifier
from sklearn.linear_model import LogisticRegression
from xgboost import XGBClassifier
from sklearn.naive_bayes import GaussianNB
from sklearn.svm import SVC
from imblearn.over_sampling import SMOTE
import matplotlib.pyplot as plt
import shap


def calculate_average_report(reports):
 def average_dict(dicts):
 keys = set().union(*(d.keys() for d in dicts))
 average = {}
 for key in keys:
 values = [d[key] for d in dicts if key in d]
 if all(isinstance(v, dict) for v in values):
 average[key] = average_dict(values)
 else:
 average[key] = np.mean(values) if values else 0
 return average

 return average_dict(reports)

### Load the data
data = pd.read_csv(' ')

###Specify X and Y
X = data.drop(' X ', axis=1).values
y = data[' Y '].values

feature_names = data.columns[:-1].tolist()


kf = KFold(n_splits=5, shuffle=True, random_state=42)

# Define the classifier and its hyperparameters
classifiers = {
 'Decision Tree': {
 'model': DecisionTreeClassifier(),
 'params': {'max_depth': [None, 5, 10, 20], 'min_samples_split': [2, 5, 10]},
 'short_name': 'DT'
 },
 'Random Forest': {
 'model': RandomForestClassifier(),
 'params': {'n_estimators': [50, 100, 200], 'max_depth': [None, 10, 20], 'min_samples_split': [2, 5, 10]},
 'short_name': 'RF'
 },
 'SVM': {
 'model': SVC(probability=True),
 'params': {'C': [0.01, 0.1, 1, 10], 'kernel': ['linear', 'rbf'], 'gamma': ['scale', 'auto']},
 'short_name': 'SVM'
 },
 'Logistic Regression': {
 'model': LogisticRegression(solver='liblinear'),
 'params': {'C': [0.01, 0.1, 1, 10], 'penalty': ['l1', 'l2']},
 'short_name': 'LR'
 },
 'XGBoost': {
 'model': XGBClassifier(use_label_encoder=False),
 'params': {'n_estimators': [50, 100, 200], 'max_depth': [3, 6, 9], 'learning_rate': [0.01, 0.1, 0.3]},
 'short_name': 'XGB'
 },
 'Naive Bayes': {
 'model': GaussianNB(),
 'params': {},
 'short_name': 'NB'
 }

}


classification_reports_not_oversampled = {name: [] for name in classifiers}
classification_reports_oversampled = {name: [] for name in classifiers}
roc_data_not_oversampled = {name: [] for name in classifiers}
roc_data_oversampled = {name: [] for name in classifiers}


for fold, (train_index, val_index) in enumerate(kf.split(X)):
 print(f'Fold {fold + 1}')

 X_train, X_val = X[train_index], X[val_index]
 y_train, y_val = y[train_index], y[val_index]


 if len(np.unique(y_val)) < 2:
 print(f"Skipping fold {fold + 1} due to single class in validation set.")
 continue


 scaler = StandardScaler()
 X_train = scaler.fit_transform(X_train)
 X_val = scaler.transform(X_val)


 smote = SMOTE(random_state=42)
 X_train_oversampled, y_train_oversampled = smote.fit_resample(X_train, y_train)

 for name, classifier_info in classifiers.items():
 model = classifier_info['model']
 params = classifier_info['params']
 short_name = classifier_info['short_name']


 grid_search = GridSearchCV(model, params, cv=5, scoring='roc_auc', n_jobs=-1)
 grid_search.fit(X_train, y_train)
 best_model = grid_search.best_estimator_


 y_pred_prob = best_model.predict_proba(X_val)[:, 1]
 report = classification_report(y_val, (y_pred_prob > 0.5).astype(int), output_dict=True, zero_division=1)
 report['AUC'] = auc(*roc_curve(y_val, y_pred_prob)[:2]) # 添加 AUC 到报告
 classification_reports_not_oversampled[name].append(report)


 fpr, tpr, _ = roc_curve(y_val, y_pred_prob)
 roc_data_not_oversampled[name].append((fpr, tpr))


 best_model.set_params(**grid_search.best_params_)
 best_model.fit(X_train_oversampled, y_train_oversampled)
 y_pred_prob_oversampled = best_model.predict_proba(X_val)[:, 1]


 if np.isnan(y_pred_prob_oversampled).any() or len(np.unique(y_pred_prob_oversampled)) == 1:
 print(f"Skipping {name} in fold {fold + 1} (oversampled) due to NaN or single class prediction.")
 continue

 report_oversampled = classification_report(y_val, (y_pred_prob_oversampled > 0.5).astype(int), output_dict=True,
 zero_division=1)
 report_oversampled['AUC'] = auc(*roc_curve(y_val, y_pred_prob_oversampled)[:2]) # 添加 AUC 到报告
 classification_reports_oversampled[name].append(report_oversampled)


 fpr_over, tpr_over, _ = roc_curve(y_val, y_pred_prob_oversampled)
 roc_data_oversampled[name].append((fpr_over, tpr_over))


mean_fpr_not_oversampled = np.linspace(0, 1, 100)
mean_tpr_not_oversampled = {name: np.zeros_like(mean_fpr_not_oversampled) for name in classifiers}
mean_auc_not_oversampled = {name: 0 for name in classifiers}
for name, roc_data in roc_data_not_oversampled.items():
 for fpr, tpr in roc_data:
 mean_tpr_not_oversampled[name] += np.interp(mean_fpr_not_oversampled, fpr, tpr)
 mean_auc_not_oversampled[name] += auc(fpr, tpr)
 mean_tpr_not_oversampled[name] /= len(roc_data)
 mean_auc_not_oversampled[name] /= len(roc_data)

mean_fpr_oversampled = np.linspace(0, 1, 100)
mean_tpr_oversampled = {name: np.zeros_like(mean_fpr_oversampled) for name in classifiers}
mean_auc_oversampled = {name: 0 for name in classifiers}
for name, roc_data in roc_data_oversampled.items():
 for fpr, tpr in roc_data:
 mean_tpr_oversampled[name] += np.interp(mean_fpr_oversampled, fpr, tpr)
 mean_auc_oversampled[name] += auc(fpr, tpr)
 mean_tpr_oversampled[name] /= len(roc_data)
 mean_auc_oversampled[name] /= len(roc_data)


custom_colors = [
 '#feb2da',
 '#d495e0',
 '#ad8fdc',
 '#8475c5',
 '#86dcf4',
 '#71bcec'
]


plt.figure(figsize=(10, 7))
lw = 2
for i, (name, info) in enumerate(classifiers.items()):
 plt.plot(mean_fpr_not_oversampled, mean_tpr_not_oversampled[name], lw=lw,
 label=f'{info["short_name"]} AUC = {mean_auc_not_oversampled[name]:.2f}',
 color=custom_colors[i])
plt.plot([0, 1], [0, 1], color='gray', linestyle='--', lw=lw)
plt.xlim([0.0, 1.0])
plt.ylim([0.0, 1.05])
plt.xlabel('False Positive Rate', fontsize=10)
plt.ylabel('True Positive Rate', fontsize=10)
plt.legend(loc='lower right')
plt.savefig('roc_not_oversampled.png')


plt.figure(figsize=(10, 7))
for i, (name, info) in enumerate(classifiers.items()):
 plt.plot(mean_fpr_oversampled, mean_tpr_oversampled[name], lw=lw,
 label=f'{info["short_name"]} AUC = {mean_auc_oversampled[name]:.2f}',
 color=custom_colors[i])
plt.plot([0, 1], [0, 1], color='gray', linestyle='--', lw=lw)
plt.xlim([0.0, 1.0])
plt.ylim([0.0, 1.05])
plt.xlabel('False Positive Rate', fontsize=10)
plt.ylabel('True Positive Rate', fontsize=10)
plt.legend(loc='lower right')
plt.savefig('roc_oversampled.png')


average_report_not_oversampled = {name: calculate_average_report(reports) for name, reports in
 classification_reports_not_oversampled.items()}
average_report_oversampled = {name: calculate_average_report(reports) for name, reports in
 classification_reports_oversampled.items()}


with pd.ExcelWriter('6classifier_classification_reports.xlsx') as writer:
 for name, info in classifiers.items():
 pd.DataFrame(average_report_not_oversampled[name]).to_excel(writer,
 sheet_name=f'Not_Oversampled_{info["short_name"]}')
 pd.DataFrame(average_report_oversampled[name]).to_excel(writer, sheet_name=f'Oversampled_{info["short_name"]}')


best_model_not_oversampled = max(classifiers, key=lambda k: mean_auc_not_oversampled[k])
best_model_oversampled = max(classifiers, key=lambda k: mean_auc_oversampled[k])


print(f"Best Model Not Oversampled: {classifiers[best_model_not_oversampled]['short_name']} ({best_model_not_oversampled})")
print(f"Best Model Oversampled: {classifiers[best_model_oversampled]['short_name']} ({best_model_oversampled})")


X_train_full = np.concatenate([X_train, X_val])
y_train_full = np.concatenate([y_train, y_val])


best_model_not_oversampled_grid_search = GridSearchCV(classifiers[best_model_not_oversampled]['model'],
 classifiers[best_model_not_oversampled]['params'],
 cv=5, scoring='roc_auc', n_jobs=-1)
best_model_not_oversampled_grid_search.fit(X_train_full, y_train_full)
best_model_not_oversampled_instance = best_model_not_oversampled_grid_search.best_estimator_

best_model_oversampled_grid_search = GridSearchCV(classifiers[best_model_oversampled]['model'],
 classifiers[best_model_oversampled]['params'],
 cv=5, scoring='roc_auc', n_jobs=-1)
best_model_oversampled_grid_search.fit(X_train_oversampled, y_train_oversampled)
best_model_oversampled_instance = best_model_oversampled_grid_search.best_estimator_


explainer_not_oversampled = shap.KernelExplainer(lambda x: best_model_not_oversampled_instance.predict_proba(x)[:, 1], X_train)
shap_values_not_oversampled = explainer_not_oversampled.shap_values(X_val)


explainer_oversampled = shap.KernelExplainer(lambda x: best_model_oversampled_instance.predict_proba(x)[:, 1], X_train_oversampled)
shap_values_oversampled = explainer_oversampled.shap_values(X_val)


def get_shap_values(explainer, X_val):
 shap_values = explainer.shap_values(X_val)
 return shap_values


plt.rcParams['font.family'] = 'Arial'
plt.rcParams['font.size'] = 7


def plot_shap_importance(shap_values, X_val, feature_names, title, filename, color):

 plt.figure(figsize=(24, 26))
 shap.summary_plot(shap_values, X_val, feature_names=feature_names, max_display=66, plot_type="bar", show=False,
 color=color)

 plt.xlabel("Mean |SHAP value| (impact on model output)", fontsize=7)
 plt.ylabel("Feature", fontsize=7)


 ax = plt.gca()

 labels = ax.get_yticklabels()
 plt.setp(labels, fontsize=7)

 labels = ax.get_xticklabels()
 plt.setp(labels, fontsize=7)

 for p in plt.gca().patches:
 plt.gca().text(p.get_x() + p.get_width() + 0.05, p.get_y() + 0.5 * p.get_height(),
 '{:.3f}'.format(p.get_width()), ha='left', va='center', fontsize=9)

 plt.title(title, fontsize=7)
 plt.tight_layout()
 plt.savefig(filename, dpi=300, bbox_inches='tight', pad_inches=0.1, format='png')
 plt.show()


plot_shap_importance(shap_values_not_oversampled, X_val, feature_names,
 title=" ",
 filename="shap_importance_not_oversampled.png",
 color="#778ccc")


plot_shap_importance(shap_values_oversampled, X_val, feature_names,
 title=" ",
 filename="shap_importance_oversampled.png",
 color="#fd8c67")


explainer = shap.KernelExplainer(lambda x: best_model_not_oversampled_instance.predict_proba(x)[:, 1], X_train)
shap_values_not_oversampled = explainer.shap_values(X_val)


plt.figure(figsize=(24, 26))

# SHAP
shap.summary_plot(shap_values_not_oversampled, X_val, feature_names=feature_names, max_display=66, plot_type="dot", show=False)
plt.xlabel("Mean |SHAP value| (impact on model output)", fontsize=7)
ax = plt.gca()

for label in ax.get_yticklabels():
 label.set_fontsize(9)

for label in ax.get_xticklabels():
 label.set_fontsize(9)


plt.tight_layout()
plt.savefig(f'shap_summary_{classifiers[best_model_not_oversampled]["short_name"]}_not_oversampled.png', dpi=300,
 bbox_inches='tight', pad_inches=0.1, format='png')
plt.show()


explainer = shap.KernelExplainer(lambda x: best_model_oversampled_instance.predict_proba(x)[:, 1], X_train_oversampled)
shap_values_oversampled = explainer.shap_values(X_val)

plt.figure(figsize=(24, 26))
shap.summary_plot(shap_values_oversampled, X_val, feature_names=feature_names, max_display=66, plot_type="dot", show=False)

plt.xlabel("Mean |SHAP value| (impact on model output)", fontsize=7)
ax = plt.gca()

for label in ax.get_yticklabels():
 label.set_fontsize(9)

for label in ax.get_xticklabels():
 label.set_fontsize(9)

plt.tight_layout()
plt.savefig(f'shap_summary_{classifiers[best_model_oversampled]["short_name"]}_oversampled.png', dpi=300,
 bbox_inches='tight', pad_inches=0.1, format='png')
plt.show()

**DNN code**

import numpy as np
import pandas as pd
from sklearn.model_selection import KFold, GridSearchCV
from sklearn.metrics import classification_report, roc_curve, auc
from sklearn.preprocessing import StandardScaler
from tensorflow.keras.models import Sequential
from tensorflow.keras.layers import Dense, Dropout
from scikeras.wrappers import KerasClassifier
from imblearn.over_sampling import SMOTE
import matplotlib.pyplot as plt
import shap

def calculate_average_report(reports):
 def average_dict(dicts):
 keys = set().union(*(d.keys() for d in dicts))
 average = {}
 for key in keys:
 values = [d[key] for d in dicts if key in d]
 if all(isinstance(v, dict) for v in values):
 average[key] = average_dict(values)
 else:
 average[key] = np.mean(values) if values else 0
 return average

 return average_dict(reports)

data = pd.read_csv(' ')
X = data.drop(' ', axis=1).values
y = data[' '].values

scaler = StandardScaler()
X = scaler.fit_transform(X)

kf = KFold(n_splits=5, shuffle=True, random_state=42)

def create_dnn(units_1, units_2, dropout_rate):
 model = Sequential()
 model.add(Dense(units_1, input_dim=X.shape[1], activation='relu'))
 model.add(Dense(units_2, activation='relu'))
 model.add(Dropout(dropout_rate))
 model.add(Dense(32, activation='relu'))
 model.add(Dense(1, activation='sigmoid'))
 model.compile(loss='binary_crossentropy', optimizer='adam', metrics=['accuracy'])
 return model


param_grid = {
 'units_1': [64, 128],
 'units_2': [32, 64],
 'dropout_rate': [0.3, 0.5],
}


model = KerasClassifier(model=create_dnn, units_1=128, units_2=64, dropout_rate=0.5, epochs=200, batch_size=10)
grid = GridSearchCV(estimator=model, param_grid=param_grid, scoring='accuracy', cv=3)
grid_result = grid.fit(X, y)

print(f": {grid_result.best_params_}")
print(f": {grid_result.best_score_}")


best_params = grid_result.best_params_
model = KerasClassifier(model=create_dnn, **best_params, epochs=5, batch_size=10)
model.fit(X, y)


classification_reports_not_oversampled = []
classification_reports_oversampled = []
roc_data_not_oversampled = []
roc_data_oversampled = []
all_shap_values_not_oversampled = []
all_shap_values_oversampled = []

for fold, (train_index, val_index) in enumerate(kf.split(X)):
 print(f'Fold {fold + 1}')
 X_train, X_val = X[train_index], X[val_index]
 y_train, y_val = y[train_index], y[val_index]

 smote = SMOTE(random_state=42)
 X_train_oversampled, y_train_oversampled = smote.fit_resample(X_train, y_train)

 model.fit(X_train, y_train, epochs=200, batch_size=10, verbose=0)
 y_pred_prob = model.predict(X_val)

 if np.any(y_val == 1) and np.any(y_val == 0):
 fpr, tpr, _ = roc_curve(y_val, y_pred_prob)
 report = classification_report(y_val, (y_pred_prob > 0.5).astype(int), output_dict=True, zero_division=1)
 report['AUC'] = auc(fpr, tpr)
 classification_reports_not_oversampled.append(report)
 roc_data_not_oversampled.append((fpr, tpr))

 model.fit(X_train_oversampled, y_train_oversampled, epochs=200, batch_size=10, verbose=0)
 y_pred_prob_oversampled = model.predict(X_val)

 if np.any(y_val == 1) and np.any(y_val == 0):
 fpr_over, tpr_over, _ = roc_curve(y_val, y_pred_prob_oversampled)
 report_oversampled = classification_report(y_val, (y_pred_prob_oversampled > 0.5).astype(int), output_dict=True, zero_division=1)
 report_oversampled['AUC'] = auc(fpr_over, tpr_over)
 classification_reports_oversampled.append(report_oversampled)
 roc_data_oversampled.append((fpr_over, tpr_over))

def calculate_mean_roc(roc_data):
 mean_fpr = np.linspace(0, 1, 100)
 mean_tpr = np.zeros_like(mean_fpr)
 mean_auc = 0
 for fpr, tpr in roc_data:
 mean_tpr += np.interp(mean_fpr, fpr, tpr)
 mean_auc += auc(fpr, tpr)
 mean_tpr /= len(roc_data)
 mean_auc /= len(roc_data)
 return mean_fpr, mean_tpr, mean_auc

mean_fpr_not_oversampled, mean_tpr_not_oversampled, mean_auc_not_oversampled = calculate_mean_roc(roc_data_not_oversampled)
mean_fpr_oversampled, mean_tpr_oversampled, mean_auc_oversampled = calculate_mean_roc(roc_data_oversampled)


plt.figure(figsize=(10, 7))
lw = 2
plt.plot(mean_fpr_not_oversampled, mean_tpr_not_oversampled, color='#d35b7e', lw=lw,
 label=f'Mean ROC (Not Oversampled) AUC = {mean_auc_not_oversampled:.2f}')
plt.plot(mean_fpr_oversampled, mean_tpr_oversampled, color='#ef836c', lw=lw,
 label=f'Mean ROC (Oversampled) AUC = {mean_auc_oversampled:.2f}')
plt.plot([0, 1], [0, 1], color='gray', linestyle='--', lw=lw)
plt.xlim([0.0, 1.0])
plt.ylim([0.0, 1.05])
plt.xlabel('False Positive Rate')
plt.ylabel('True Positive Rate')
plt.legend(loc='lower right')
plt.savefig('average_roc.png')
plt.show()


average_report_not_oversampled = calculate_average_report(classification_reports_not_oversampled)
average_report_oversampled = calculate_average_report(classification_reports_oversampled)


with pd.ExcelWriter('dnnclassification_reports.xlsx') as writer:
 pd.DataFrame(average_report_not_oversampled).to_excel(writer, sheet_name='Not Oversampled')
 pd.DataFrame(average_report_oversampled).to_excel(writer, sheet_name='Oversampled')


explainer = shap.KernelExplainer(lambda x: model.predict(x).flatten(), X_train)
shap_values_not_oversampled = explainer.shap_values(X_val)
all_shap_values_not_oversampled.append(shap_values_not_oversampled)

explainer_oversampled = shap.KernelExplainer(lambda x: model.predict(x).flatten(), X_train_oversampled)
shap_values_oversampled = explainer_oversampled.shap_values(X_val)
all_shap_values_oversampled.append(shap_values_oversampled)

feature_names_list = data.columns[:-1].tolist()
feature_names = feature_names_list


plt.rcParams['font.family'] = 'Arial'
plt.rcParams['font.size'] = 7


def plot_shap_importance(shap_values, X_val, feature_names, title, filename, color):
 plt.figure(figsize=(24, 26))
 shap.summary_plot(shap_values, X_val, feature_names=feature_names, max_display=66, plot_type="bar", show=False,
 color=color)


 plt.xlabel("Mean |SHAP value| (impact on model output)", fontsize=7)
 plt.ylabel("Feature", fontsize=7)


 ax = plt.gca()


 labels = ax.get_yticklabels()
 plt.setp(labels, fontsize=7)

 labels = ax.get_xticklabels()
 plt.setp(labels, fontsize=7)


 for p in plt.gca().patches:
 plt.gca().text(p.get_x() + p.get_width() + 0.05, p.get_y() + 0.5 * p.get_height(),
 '{:.3f}'.format(p.get_width()), ha='left', va='center', fontsize=7)

 plt.title(title, fontsize=7)
 plt.tight_layout()
 plt.savefig(filename, dpi=300, bbox_inches='tight', pad_inches=0.1, format='png')
 plt.show()


plot_shap_importance(shap_values_not_oversampled, X_val, feature_names,
 title=" ",
 filename="shap_importance_not_oversampled.png",
 color="#A86A9D")

plot_shap_importance(shap_values_oversampled, X_val, feature_names,
 title=" ",
 filename="shap_importance_oversampled.png",
 color="#FBCB1F")


avg_shap_values_not_oversampled = np.mean(all_shap_values_not_oversampled, axis=0)
feature_names_list = data.columns[:-1].tolist()
plt.figure(figsize=(24, 26))

shap.summary_plot(shap_values_not_oversampled, X_val, feature_names=feature_names, max_display=66, plot_type="dot", show=False)

plt.xlabel("Mean |SHAP value| (impact on model output)", fontsize=7)
ax = plt.gca()
for label in ax.get_yticklabels():
 label.set_fontsize(7)
for label in ax.get_xticklabels():
 label.set_fontsize(7)

plt.tight_layout()
plt.savefig(f'shap_summary_not_oversampled.png', dpi=300,
 bbox_inches='tight', pad_inches=0.1, format='png')
plt.show()


avg_shap_values_oversampled = np.mean(all_shap_values_oversampled, axis=0)
plt.figure(figsize=(24, 26))
shap.summary_plot(shap_values_oversampled, X_val, feature_names=feature_names, max_display=66, plot_type="dot", show=False)
plt.xlabel("Mean |SHAP value| (impact on model output)", fontsize=7)
ax = plt.gca()
for label in ax.get_yticklabels():
 label.set_fontsize(7)
for label in ax.get_xticklabels():
 label.set_fontsize(7)
plt.tight_layout()
plt.savefig(f'shap_summary_oversampled.png', dpi=300,
 bbox_inches='tight', pad_inches=0.1, format='png')
plt.show()
