# Supplementary material for: Developing SHAP interpretable machine learning models for assessing biopsychosocial risk in female drug users: a small sample study
Source: Front Psychiatry. 2026 Apr 22;17:1736274. doi: 10.3389/fpsyt.2026.1736274 (PMC13148034; doi:10.3389/fpsyt.2026.1736274)
Supplement: Supplementary file 2 [file SupplementaryFile2.docx]

***Supplementary Material***

# 1 Results before oversampling

***Table S1*** *Classifier indicator results*

|  |  | Accuracy | Total precision rate | Total recall rate | Total F1 value | AUC |
| --- | --- | --- | --- | --- | --- | --- |
| The risk of physiological function | DT | 0.69 | 0.65 | 0.51 | 0.50 | 0.58 |
|  | RF | 0.63 | 0.77 | 0.54 | 0.50 | 0.75 |
|  | SVM | 0.77 | 0.89 | 0.50 | 0.44 | 0.42 |
|  | LR | 0.75 | 0.78 | 0.49 | 0.43 | 0.48 |
|  | XGBoost | 0.70 | 0.60 | 0.47 | 0.43 | 0.51 |
|  | NB | 0.28 | 0.62 | 0.53 | 0.26 | 0.53 |
| The risk of psychological and cognitive function | DT | 0.60 | 0.52 | 0.52 | 0.52 | 0.57 |
|  | RF | 0.68 | 0.66 | 0.55 | 0.52 | 0.60 |
|  | SVM | 0.65 | 0.71 | 0.48 | 0.39 | 0.63 |
|  | LR | 0.71 | 0.69 | 0.61 | 0.61 | 0.64 |
|  | XGBoost | 0.68 | 0.62 | 0.54 | 0.50 | 0.62 |
|  | NB | 0.73 | 0.71 | 0.67 | 0.66 | 0.71 |
| The risk of drug dependence | DT | 0.80 | 0.67 | 0.59 | 0.57 | 0.54 |
|  | RF | 0.84 | 0.88 | 0.56 | 0.55 | 0.62 |
|  | SVM | 0.83 | 0.92 | 0.50 | 0.45 | 0.73 |
|  | LR | 0.83 | 0.82 | 0.53 | 0.50 | 0.66 |
|  | XGBoost | 0.83 | 0.77 | 0.54 | 0.53 | 0.68 |
|  | NB | 0.64 | 0.55 | 0.47 | 0.44 | 0.59 |
| The risk of social support | DT | 0.51 | 0.48 | 0.47 | 0.46 | 0.49 |
|  | RF | 0.50 | 0.46 | 0.44 | 0.44 | 0.48 |
|  | SVM | 0.58 | 0.64 | 0.52 | 0.45 | 0.56 |
|  | LR | 0.53 | 0.58 | 0.46 | 0.41 | 0.45 |
|  | XGBoost | 0.54 | 0.49 | 0.49 | 0.48 | 0.50 |
|  | NB | 0.51 | 0.42 | 0.46 | 0.43 | 0.48 |
| The risk of self-control | DT | 0.83 | 0.77 | 0.62 | 0.63 | 0.68 |
|  | RF | 0.80 | 0.81 | 0.49 | 0.44 | 0.64 |
|  | SVM | 0.81 | 0.81 | 0.49 | 0.45 | 0.53 |
|  | LR | 0.82 | 0.91 | 0.50 | 0.45 | 0.58 |
|  | XGBoost | 0.83 | 0.87 | 0.55 | 0.54 | 0.48 |
|  | DT | 0.83 | 0.77 | 0.62 | 0.63 | 0.68 |

A. ROC plot of the risk of physiological function


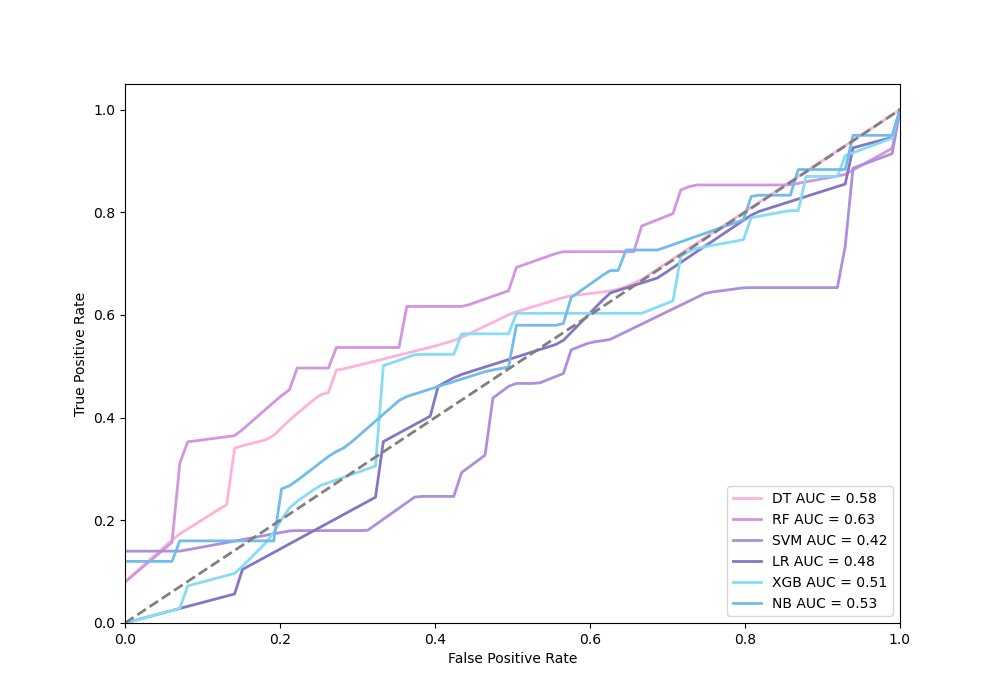


B. ROC plot of the risk of psychological and cognitive risk


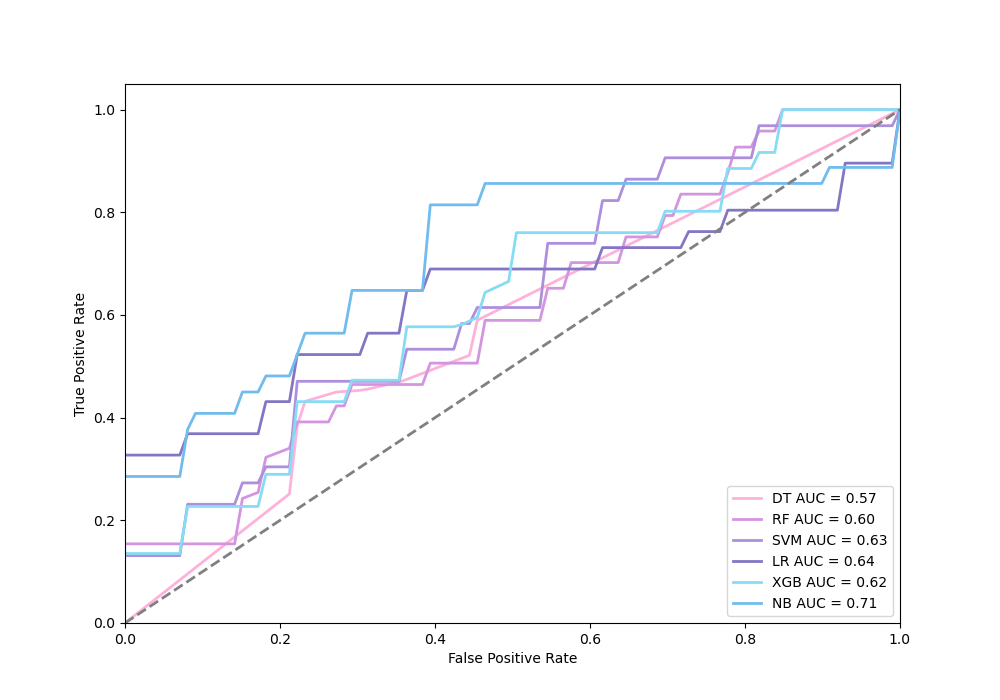

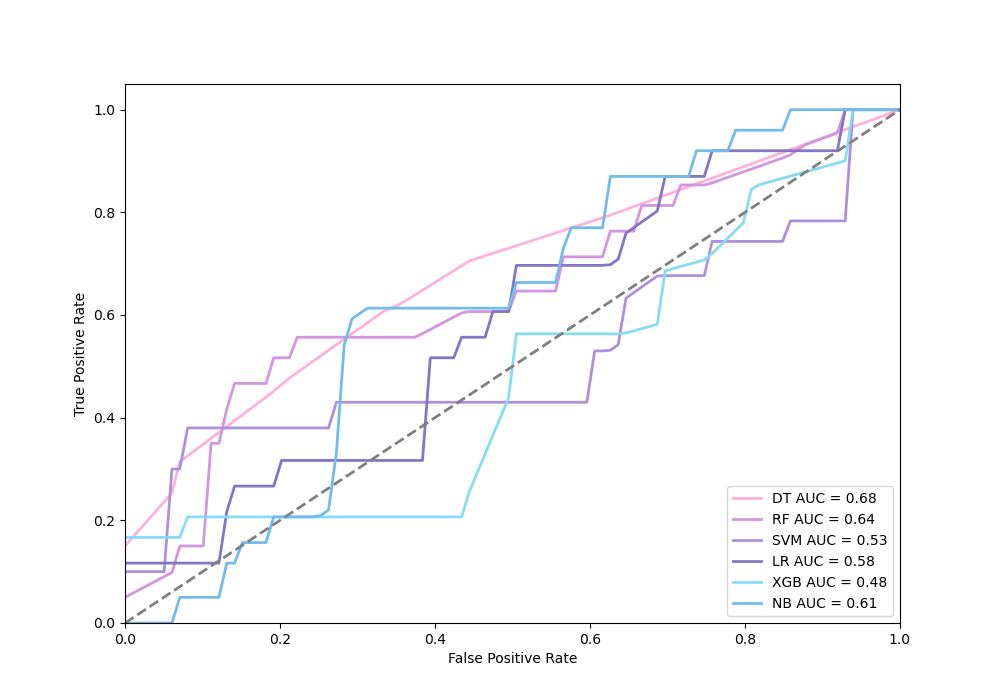


E. ROC plot of the risk of self-control


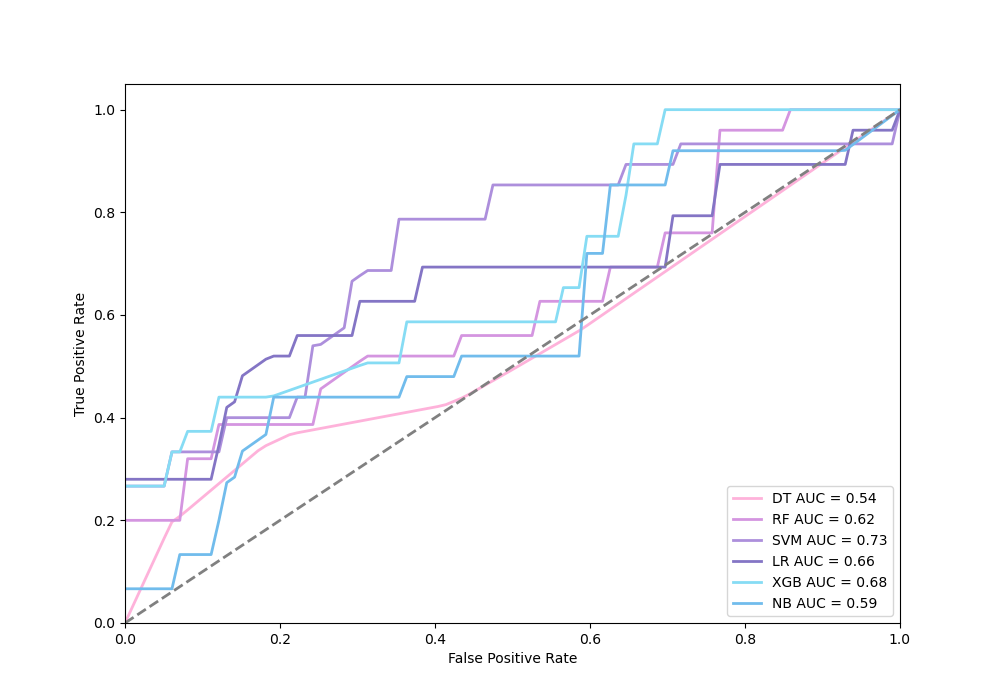

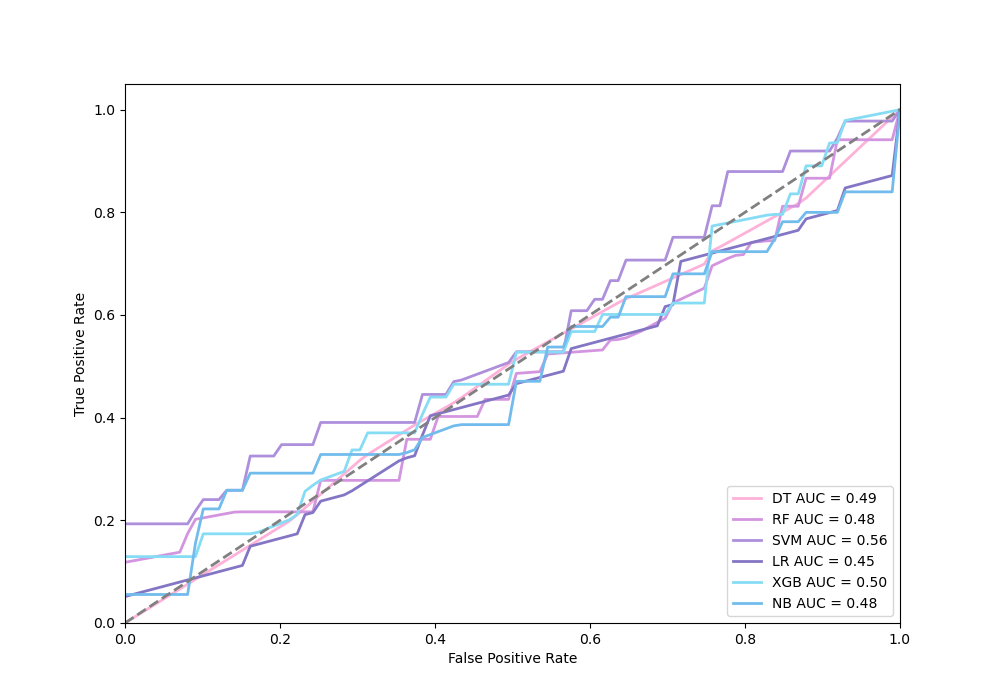


C. ROC plot of the risk of drug dependence

D. ROC plot of the risk of social support

**Supplementary Figure1**. *ROC curves for each dimension risk before oversampling*

## 1.1 The Risk of Physiological Function

**Classification Metrics.** When predicting the risk to physiological function, the accuracy ranged from 0.28 to 0.77, the total precision ranged from 0.60 and 0.89, the total recall ranged from 0.47 to 0.54, the F1 score fluctuated between 0.26 and 0.50, and the AUC value ranged from 0.42 to 0.75 (see Table S1). The ROC curves for all classifiers were presented in Supplementary Figure1A. The classifier’s performance was assessed based on key indicators, with the AUC value being the primary criterion. Among the classifiers evaluated, RF emerged as the best performer. All indicators of the RF were between 0.50 and 0.75, and the model performed well.

**Feature Importance.** The distribution of SHAP values for the 7 features predicting physiological function risk was illustrated in Supplementary Figure2A. The attribute values of the samples were depicted by the color of the points (red indicating high values and blue indicating low values; this color scheme was consistent across subsequent figures). As shown in Supplementary Figure2A, the variable "Chronic Diseases" had the most significant impact on the risk of physiological dysfunction. This suggested that the higher the likelihood of "Chronic Diseases" among female drug users, the greater the likelihood of experiencing physiological dysfunction. The rankings of feature importance were based on the average absolute SHAP values of the attributes, with higher values indicating greater importance (see Supplementary Figure2B). The most influential variable was "Chronic Diseases," which made the greatest contribution to predicting physiological risk.


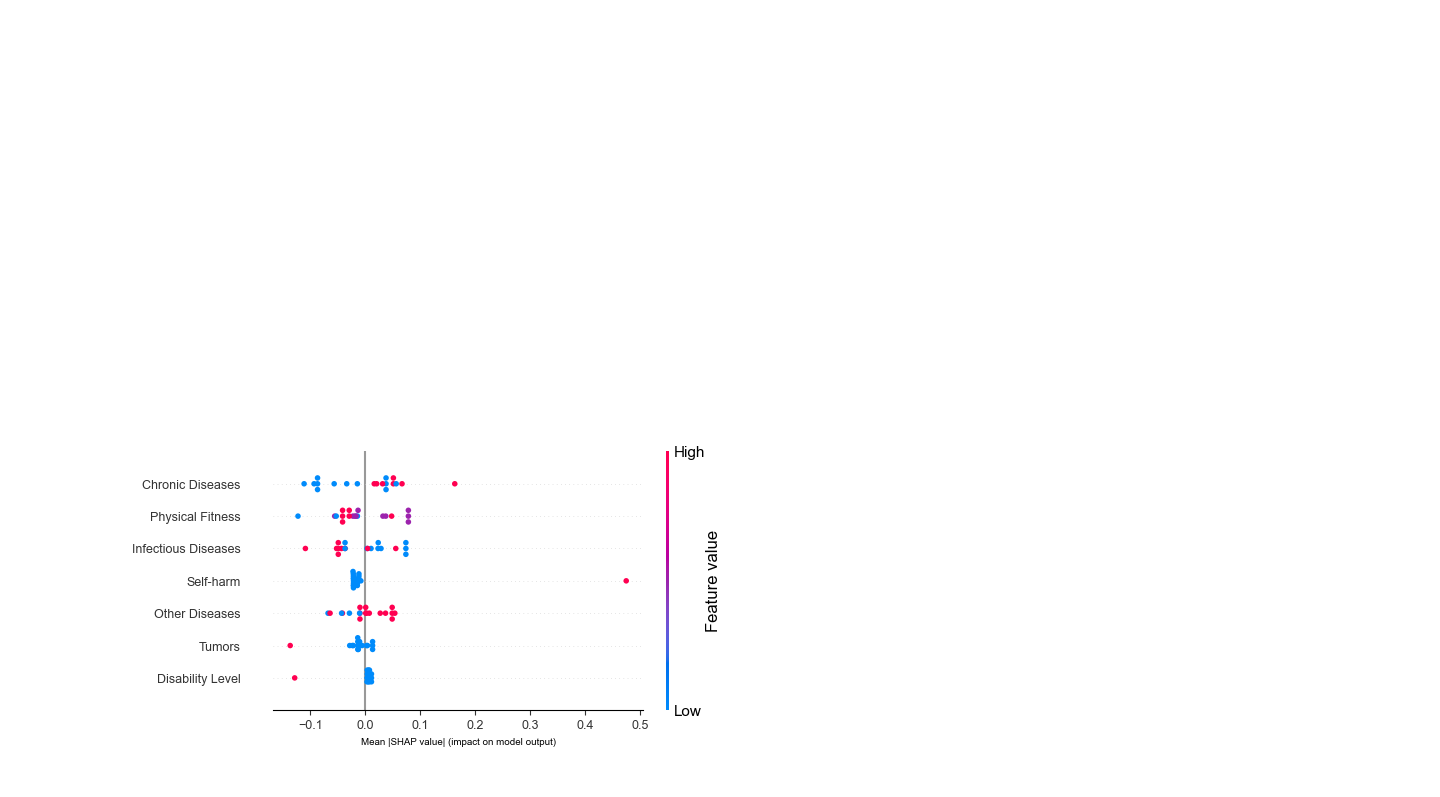


A. Distribution of SHAP values of all samples.


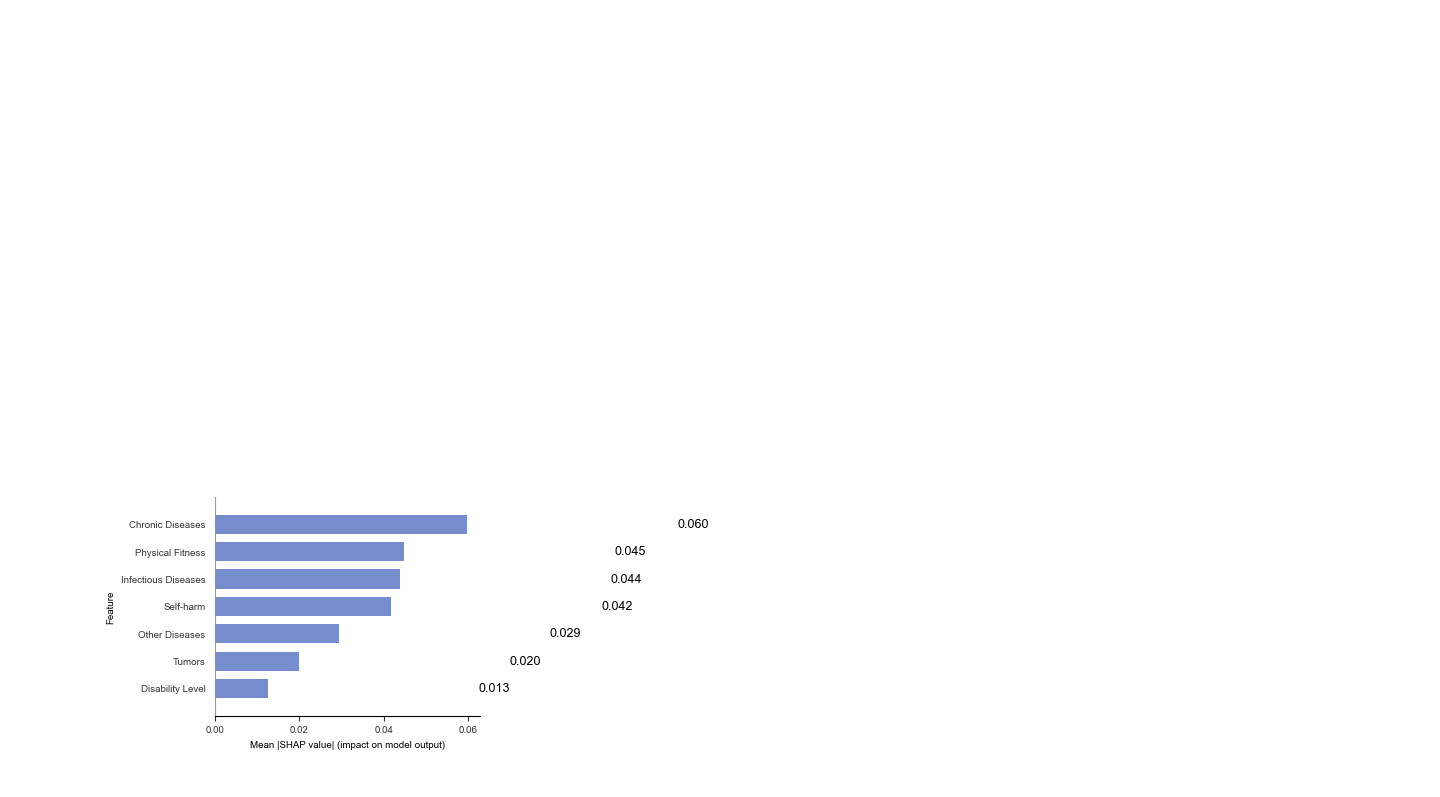


B. Ranking of the absolute value of SHAP value of all features

**Supplementary Figure2**. *SHAP results for the risk of physiological function*

## 1.2 The Risk of Psychological and Cognitive Function

**Classification Metrics.** When predicting the risk of psychological and cognitive function, the accuracy ranged from 0.61 to 0.73, the total precision ranged from 0.53 to 0.71, the total recall ranged from 0.52 to 0.67, the F1 score ranged from 0.39 and 0.66, and the AUC value ranged from 0.57 to 0.71 (see Table S1). The ROC curves for all classifiers were presented in Supplementary Figure1B. The classifier’s performance was evaluated based on key indicators, with the AUC value being the primary criterion. It was found that the best performer was NB. All indicators of the NB were between 0.66 and 0.73, and the model performed well.

**Feature Importance**. The distribution of SHAP values corresponding to the 31 variables predicting the risk of psychological and cognitive function was shown in the Supplementary Figure3A. According to Supplementary Figure3A, the "SCL-90" reverse score had the greatest impact on psychological and cognitive function risk, indicating that a higher "SCL-90" reverse score increased the likelihood of psychological and cognitive dysfunction. The red points corresponding to the "SCL-90" reverse score were mostly distributed on the left, while the blue points were distributed on the right, suggesting that higher values of the reverse-scored "SCL-90" were associated with negative SHAP values. In other words, higher "SCL-90" scores were linked to a higher risk of psychological and cognitive issues. The "16PF" score had the second greatest impact on this risk. It evaluates personality stability with a focus on factors such as emotional stability, resilience to stress, and self-discipline, which are critical indicators of mental health. The four cognitive training items—"Cumulative off-target", "Accuracy B", "Cumulative time elapsed", and "Cumulative on-target"—ranked 3rd to 6th in terms of their influence on the risk. Among these, "Cumulative off-target", "Cumulative time elapsed", and "Cumulative on-target" evaluated the attentional allocation and coordination abilities of female drug users. Higher scores in these areas indicated greater cognitive risk. "Accuracy B" assessed attention levels in female drug users, and the red dots for this score were positioned on the left, indicating that higher attention levels corresponded to lower cognitive risk. When the average absolute SHAP values for features were plotted, it was found that these 6 variables were the most significant contributors to explaining the risk of psychological and cognitive dysfunction (see Supplementary Figure3B).


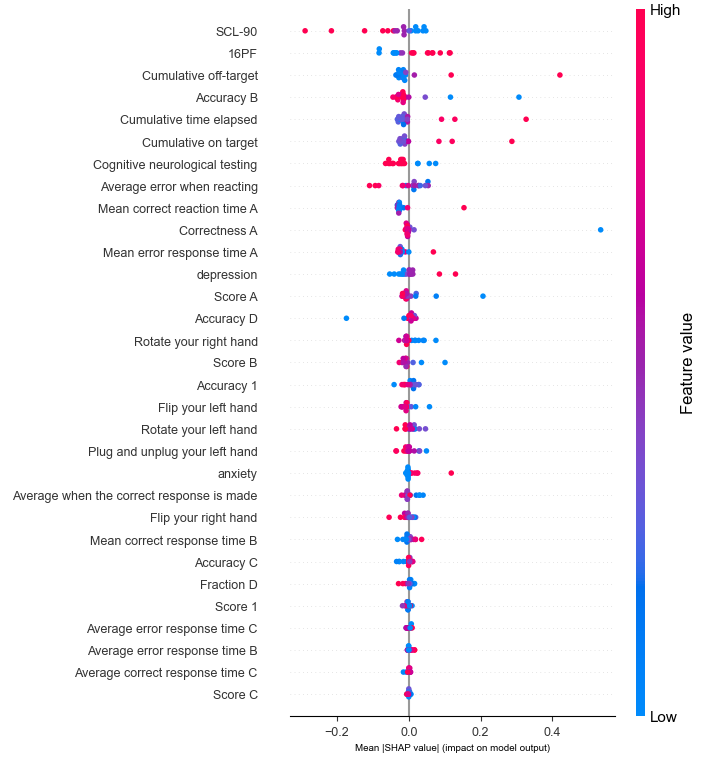


A. Distribution of SHAP values of all samples.


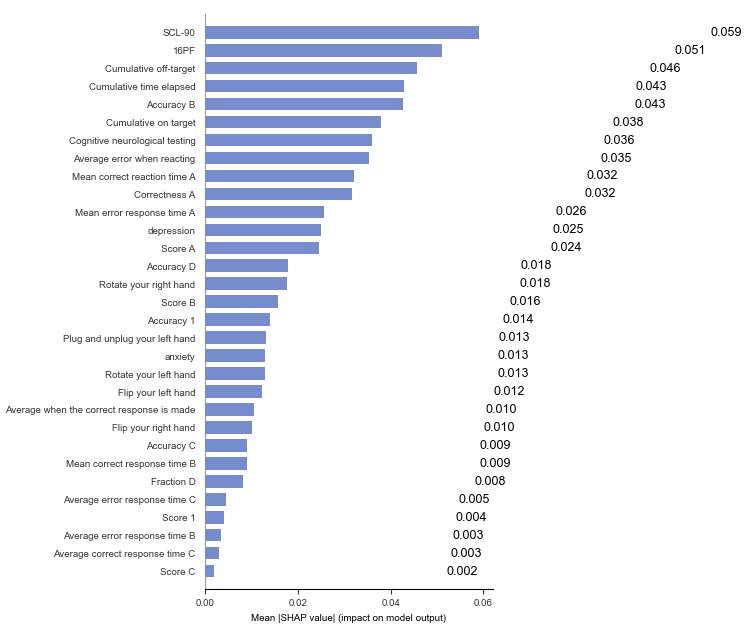


B. Ranking of the absolute value of SHAP value of all features

**Supplementary Figure3**. *SHAP results for the risk of psychological and cognitive function*

## 1.3 The risk of Drug Dependence

**Classification Metrics.** When the risk of drug dependence, the accuracy ranged from 0.64 to 0.84, the total precision ranged from 0.55 to 0.92, the total recall ranged from 0.47 to 0.59, the total F1 score ranged from 0.44 to 0.57, and the AUC value ranged from 0.54 to 0.73 (see Table S1). The ROC curves for all classifiers were shown in Supplementary Figure1C. Based on the AUC value as a key indicator, it was found that the SVM performed the best. All of SVM's indicators ranged from 0.45 to 0.92, and the model performed well.

**Feature Importance.** The distribution of SHAP values corresponding to the 6 variables predicting the risk of drug dependence was shown in the S4A figure. According to the Supplementary Figure4A, the "duration of drug use" had the greatest impact on the risk of drug dependence. Many red dots of the "duration of drug use" were distributed on the left, while blue dots were distributed on the right, indicating that the higher the duration of drug use, the corresponding SHAP value was negative. The results showed that the longer the duration of drug use for female drug users, the smaller the probability of drug dependence risk occurring, and vice versa. When the average absolute SHAP values for features were plotted, it was found that variable "duration of drug use" was the most significant contributors to explaining the risk of drug dependence (see Supplementary Figure4B).


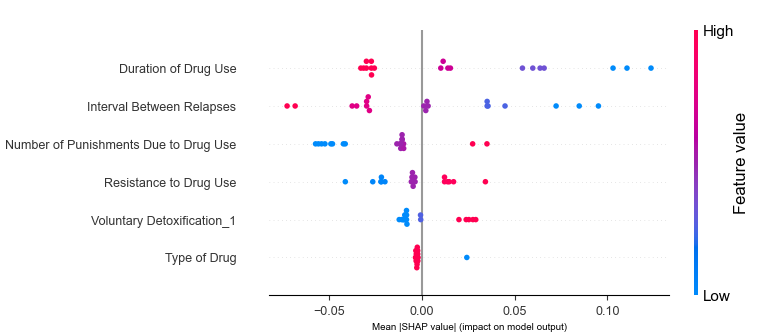


A. Distribution of SHAP values of all samples.


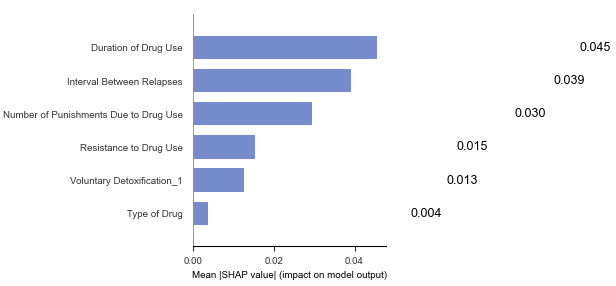


B. Ranking of the absolute value of SHAP value of all features

**Supplementary Figure4**. *SHAP results for the risk of drug dependence*

## 1.4 The Risk of Social Support

**Classification Metrics.** When predicting the risk of social support, the accuracy ranged from 0.50 and 0.58, the total precision ranged from 0.42 and 0.64, the total recall ranged from 0.44 and 0.52, the total F1 score ranged from 0.41 and 0.48, and the AUC ranged from 0.45 and 0.56(see Table S1). The ROC curves for all classifiers were shown in Supplementary Figure1D. The classifier’s performance was assessed based on key indicators, with the AUC value being the primary criterion. It was found that the best performer was SVM. All SVM indicators were between 0.45 and 0.64, and the model indicators were acceptable.

**Feature Importance.** The distribution of SHAP values corresponding to the 18 variables that predict the risk of social support was shown in the S5A figure. According to the Supplementary Figure5A, "Vocational Skills Acquired While Incarcerated" had the greatest impact on the risk of social support. The red scatter points for this feature were on the left, while the blue scatter points were on the right, indicating that the higher the level of "Vocational Skills Acquired While Incarcerated," the more likely the corresponding SHAP value was negative. In other words, a higher level of vocational skills was associated with a lower risk of needing social support for female drug users, and vice versa. Similarly, the red dots for "Educational Background Before Incarceration," "Unemployment Insurance," and "Employment Situation Before Release," ranked 2nd to 4th in impact, were also mostly distributed on the left side of the graph. This distribution suggested that a higher educational background, more stable employment prior to release, and the availability of unemployment insurance were associated with a reduced risk of requiring social support. By analyzing the average absolute SHAP values, as shown in Supplementary Figure5B, it became clear that these 4 variables had been crucial in explaining the risk of social support. Their significance highlighted the importance of considering these factors when assessing and addressing the social support needs of female drug users in the past.


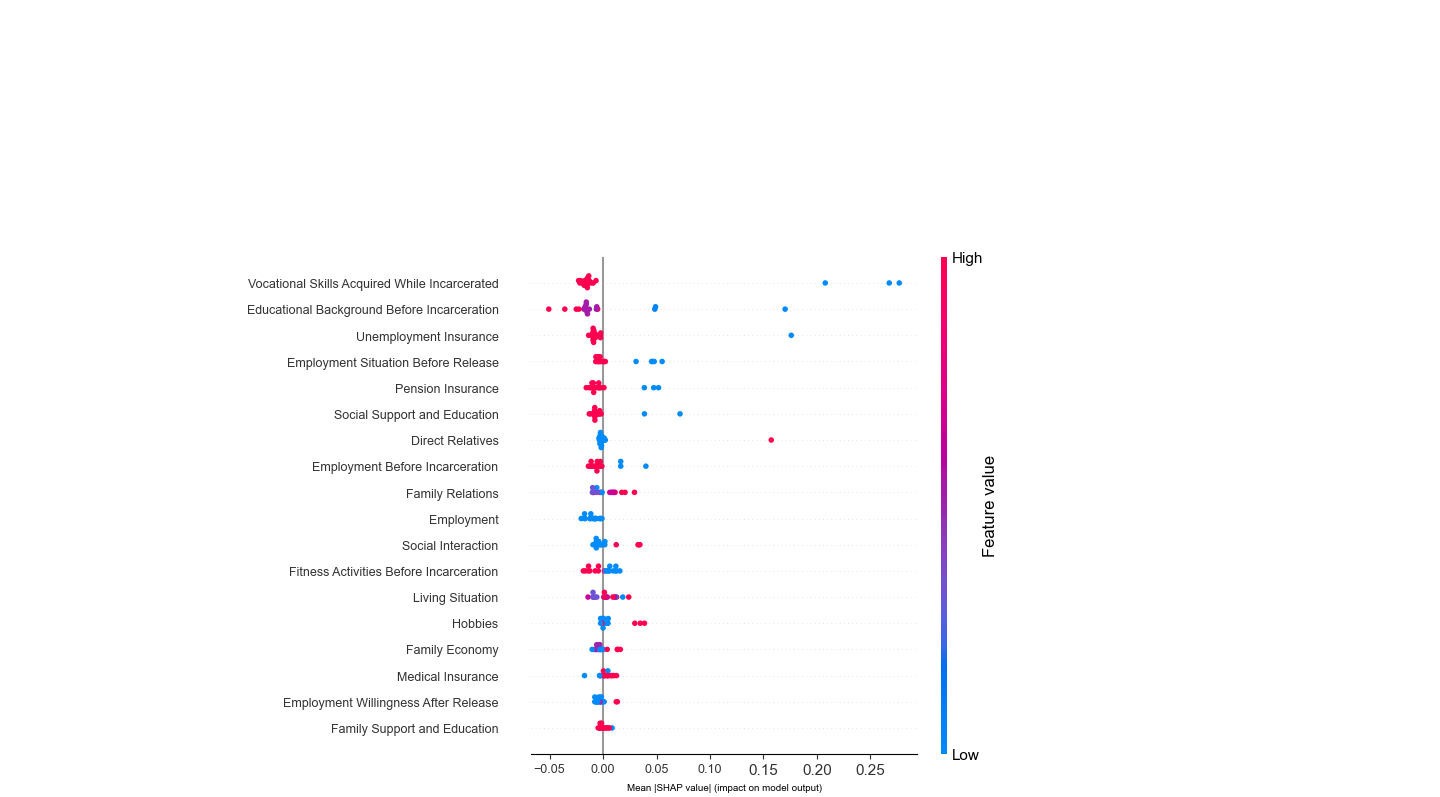


A. Distribution of SHAP values of all samples.


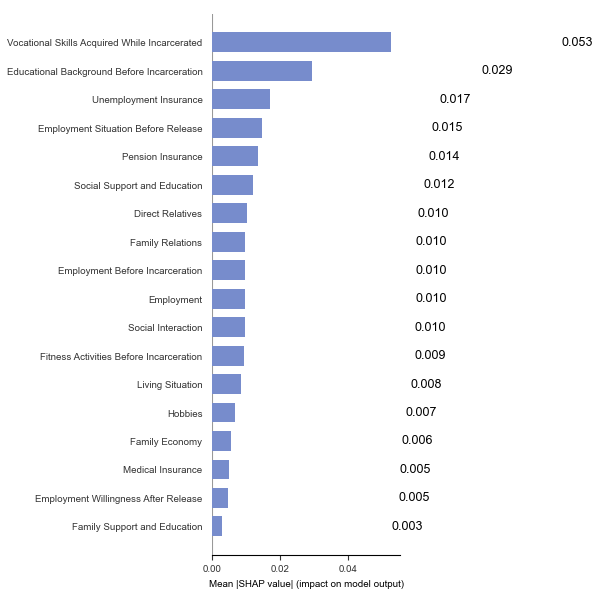


B. Ranking of the absolute value of SHAP value of all features

**Supplementary Figure5**. *SHAP results for the risk of social support*

## 1.5 The Self-Control Risk

**Classification Metrics.** When predicting the self-control risk, the accuracy ranged from 0.74 and 0.83, the total precision ranged from 0.77 and 0.91, the total recall ranged from 0.49 and 0.62, the total F1 score ranged from 0.44 and 0.63, and the AUC ranged from 0.53 and 0.68(see Table S1). The ROC curves for all classifiers were shown in Supplementary Figure1E. The performance of the classifier was selected by the key indicators of AUC value, and it was found that the best performer was DT. All indicators of DT were between 0.63 and 0.83, and the model performed well.

**Feature Importance.** The distribution of SHAP values corresponding to the 4 variables that predict the self-control risk was shown in the S6A figure. According to the S6A figure, "Compliance and Discipline" had the greatest impact on the self-control risk, indicating that the higher the level of "Compliance and Discipline" for female drug users, the greater the probability that the self-control risk appeared. Ranking according to the average absolute SHAP value of the features, as shown in Supplementary Figure6B, the first variable was "Compliance and Discipline", that was, Compliance and Discipline had the greatest contribution to the self-control risk prediction.


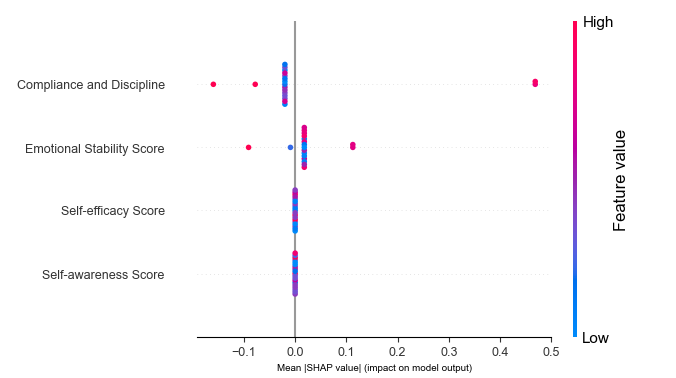


A. Distribution of SHAP values of all samples.


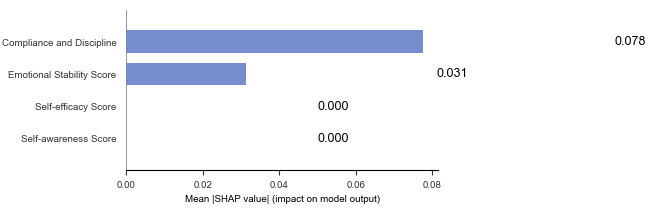


B. Ranking of the absolute value of SHAP value of all features

**Supplementary Figure6**. *SHAP results for the risk of self-control*

# 2 Results after oversampling

***Table S2*** *Classifier indicator results after oversampling*

|  |  | Accuracy | Total precision rate | Total recall rate | Total F1 value | AUC |
| --- | --- | --- | --- | --- | --- | --- |
| The risk of physiological function | DT | 0.72 | 0.62 | 0.61 | 0.61 | 0.57 |
|  | RF | 0.75 | 0.67 | 0.63 | 0.64 | 0.65 |
|  | SVM | 0.68 | 0.63 | 0.55 | 0.52 | 0.53 |
|  | LR | 0.54 | 0.54 | 0.54 | 0.50 | 0.44 |
|  | XGBoost | 0.70 | 0.59 | 0.58 | 0.58 | 0.57 |
|  | NB | 0.28 | 0.62 | 0.53 | 0.26 | 0.55 |
| The risk of psychological and cognitive function | DT | 0.51 | 0.48 | 0.48 | 0.47 | 0.48 |
|  | RF | 0.72 | 0.79 | 0.60 | 0.57 | 0.68 |
|  | SVM | 0.46 | 0.67 | 0.51 | 0.35 | 0.32 |
|  | LR | 0.69 | 0.63 | 0.63 | 0.63 | 0.67 |
|  | XGBoost | 0.67 | 0.58 | 0.58 | 0.57 | 0.68 |
|  | NB | 0.70 | 0.65 | 0.67 | 0.65 | 0.67 |
| The risk of drug dependence | DT | 0.69 | 0.53 | 0.51 | 0.51 | 0.50 |
|  | RF | 0.78 | 0.79 | 0.78 | 0.77 | 0.58 |
|  | SVM | 0.62 | 0.49 | 0.51 | 0.47 | 0.57 |
|  | LR | 0.66 | 0.60 | 0.66 | 0.57 | 0.66 |
|  | XGBoost | 0.73 | 0.55 | 0.55 | 0.54 | 0.59 |
|  | NB | 0.56 | 0.51 | 0.54 | 0.46 | 0.62 |
| The risk of social support | DT | 0.59 | 0.55 | 0.54 | 0.54 | 0.48 |
|  | RF | 0.49 | 0.39 | 0.42 | 0.40 | 0.54 |
|  | SVM | 0.46 | 0.55 | 0.46 | 0.34 | 0.43 |
|  | LR | 0.51 | 0.44 | 0.44 | 0.43 | 0.44 |
|  | XGBoost | 0.55 | 0.51 | 0.51 | 0.50 | 0.55 |
|  | NB | 0.46 | 0.37 | 0.41 | 0.38 | 0.45 |
| The risk of self-control | DT | 0.72 | 0.67 | 0.54 | 0.53 | 0.59 |
|  | RF | 0.73 | 0.69 | 0.54 | 0.54 | 0.61 |
|  | SVM | 0.65 | 0.56 | 0.45 | 0.45 | 0.48 |
|  | LR | 0.52 | 0.50 | 0.48 | 0.43 | 0.57 |
|  | XGBoost | 0.66 | 0.54 | 0.61 | 0.54 | 0.58 |
|  | NB | 0.55 | 0.53 | 0.60 | 0.48 | 0.60 |

A. ROC plot of the risk of physiological function


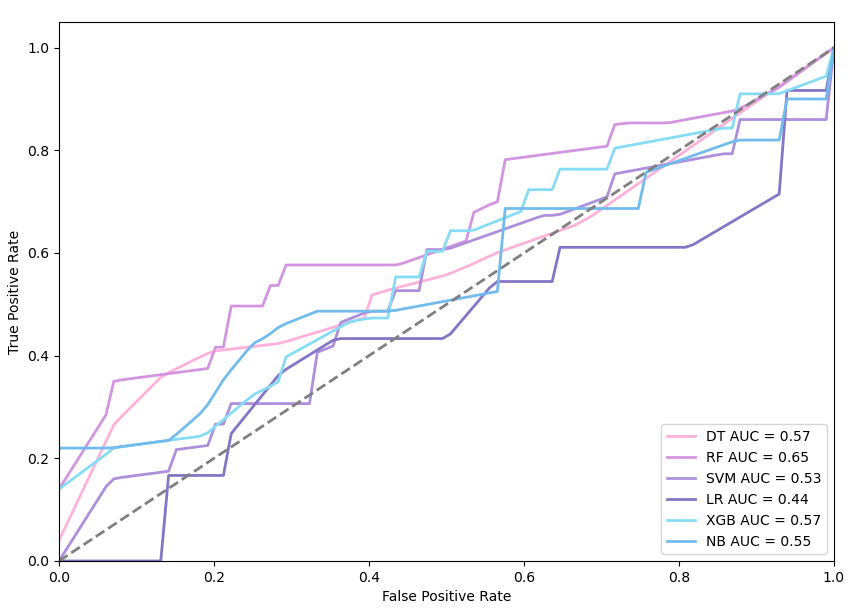


B. ROC plot of the risk of psychological and cognitive risk

E. ROC plot of the risk of self-control

C. ROC plot of the risk of drug dependence

D. ROC plot of the risk of social support


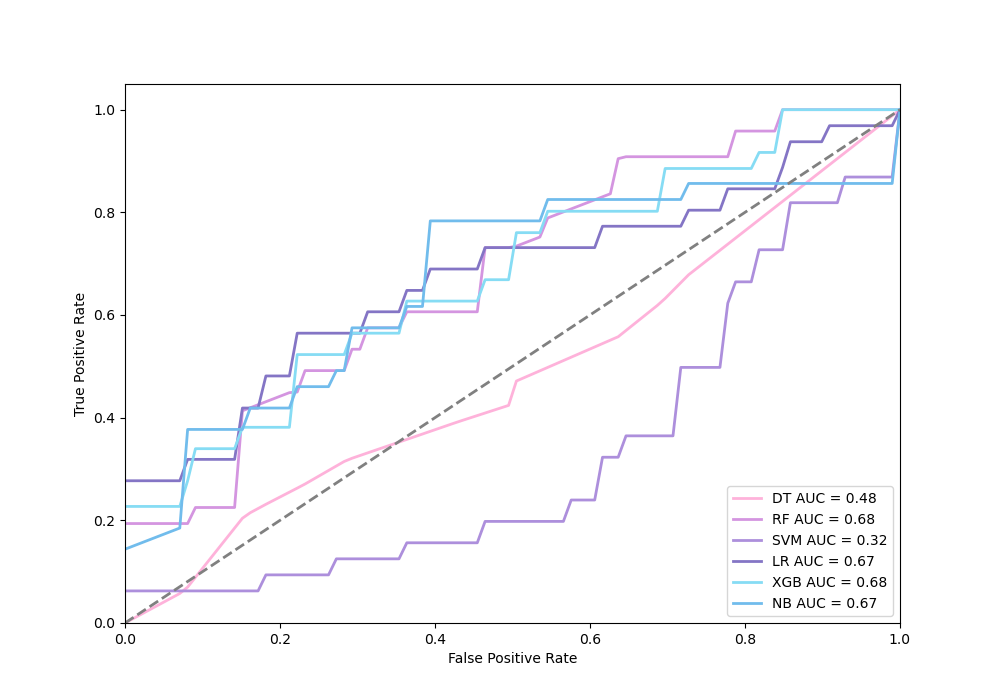

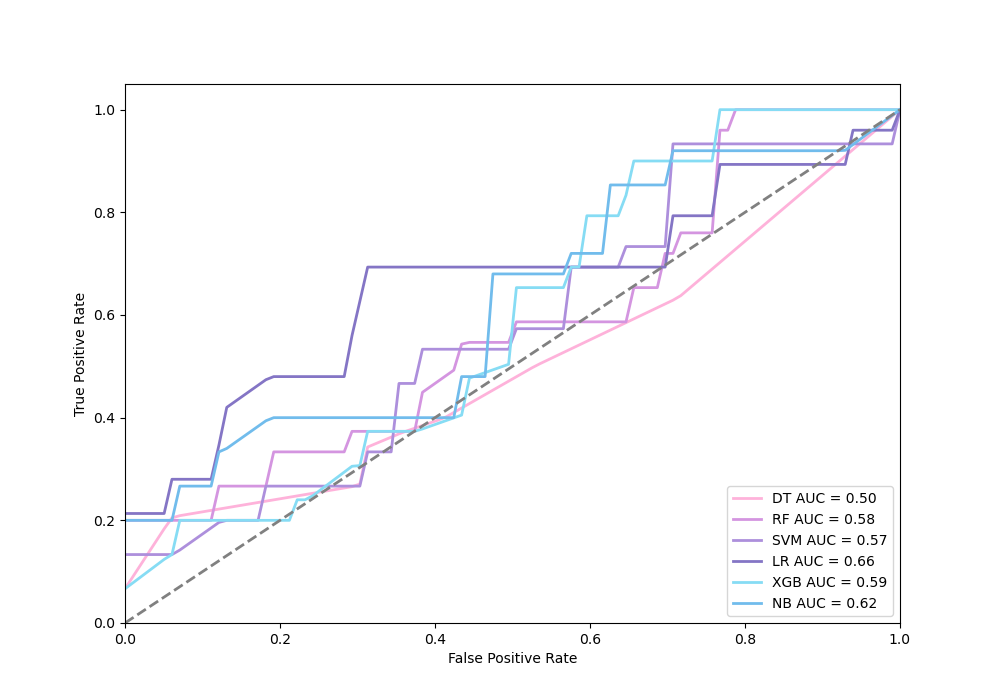

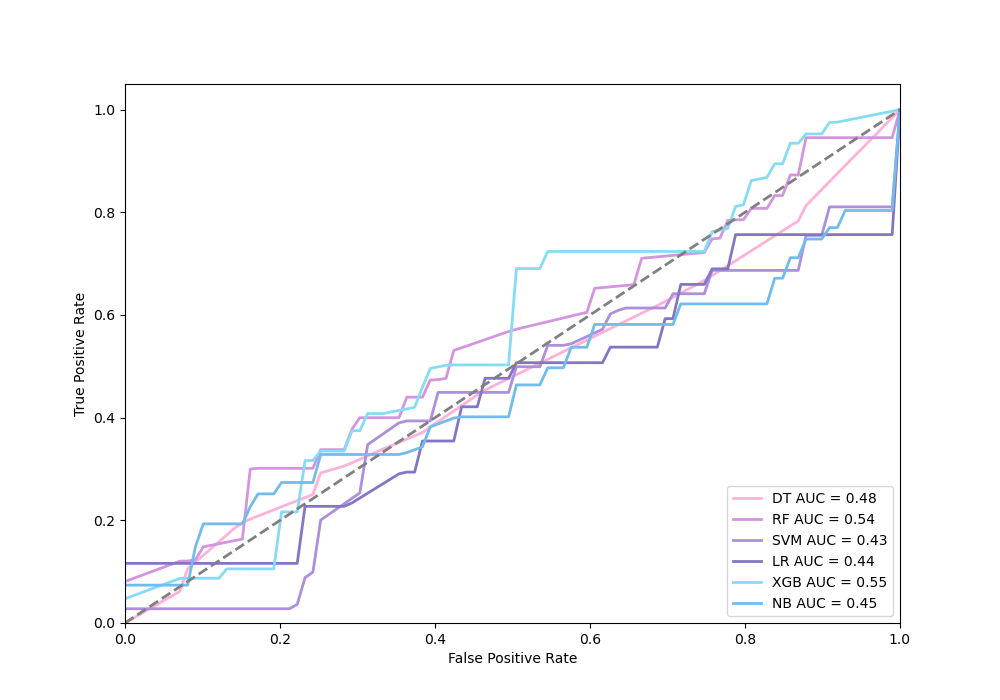

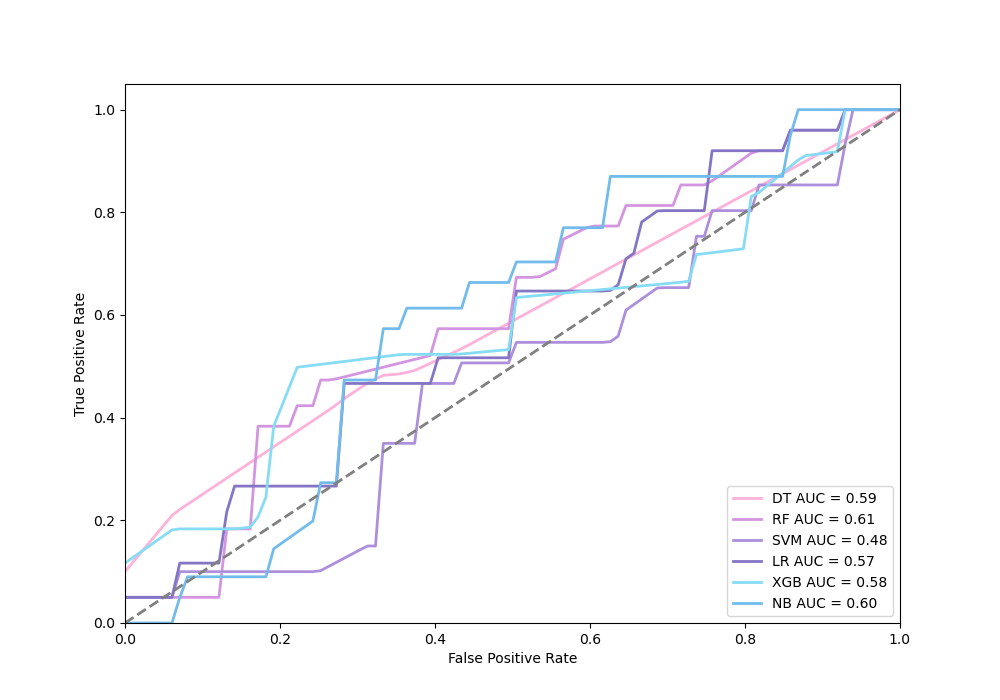


**Supplementary Figure7**. *ROC curves for each dimension risk after oversampling*

## 2.1 The Risk of Physiological Function

**Classification Metrics.** When predicting the risk of physiological function, the accuracy ranged from 0.28 to 0.75, the total precision ranged from 0.54 to 0.67, the total recall ranged from 0.53 to 0.63, the total F1 score ranged from 0.26 to 0.64, and the AUC value ranged from 0.44 to 0.65(see Table 5). The ROC curves of the classifiers were shown in Supplementary Figure7A. The performance of the classifier was selected by the key indicators of AUC value, and it was found that the best performer was RF. All indicators of the RF were between 0.63 and 0.75, and the model performed well.

**Feature Importance.** The distribution of SHAP values corresponding to the 7 variables predicting the risk of physiological function was shown in the S8A figure. According to Supplementary Figure8A, the impact of the "Physical Fitness "variable on the risk of physiological function was the greatest, suggesting that the higher the probability of "Physical Fitness" for female drug users, the more likely the risk of physiological function was to occur. Rankings were based on the average absolute SHAP value of the traits, which reflected the importance of the features (Supplementary Figure8B). The top variable had the greatest impact, the first variable being "Physical Fitness", that was, "Physical Fitness" had the greatest contribution to physiological risk prediction.


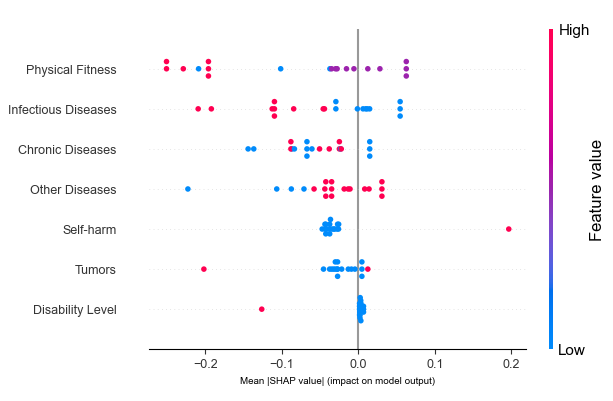


A. Distribution of SHAP values of all samples


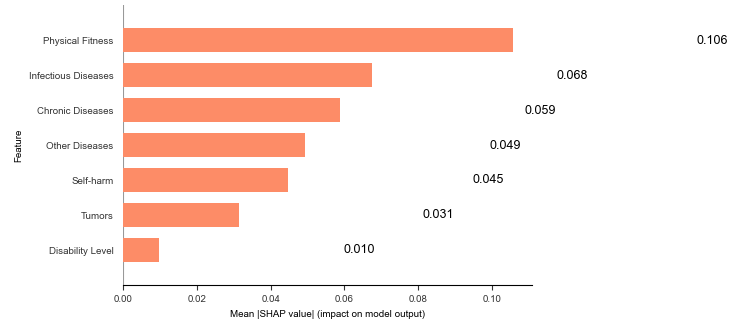


B. Ranking of the absolute value of SHAP value of all features

**Supplementary Figure8**. *SHAP results for the risk of physiological risk function after oversampling*

## 2.2 The Risk of Psychological and Cognitive Function

**Classification Metrics.** When predicting the risk of psychological and cognitive function, the accuracy ranged from 0.46 to 0.72, the total precision ranged from 0.48 to 0.79, the total recall ranged from 0.48 to 0.67, the total F1 score ranged from 0.35 to 0.65, and the AUC value ranged from 0.32 to 0.68(see Table 5). The ROC curves of the classifiers were shown in Supplementary Figure7B. The performance of the classifier was selected by the key indicators of AUC value, and it was found that the best performer was NB. All indicators of the NB were between 0.66 and 0.73, and the 7model performed well.

**Feature Importance**. The distribution of SHAP values corresponding to the 31 variables predicting the risk of psychological and cognitive function was shown in the S9A figure. According to the S9A figure, the "Cumulative off-target" had the greatest impact on the risk of psychological and cognitive skills, indicating that the higher the score of the "Cumulative off-target" of female drug users, the more likely the risk of psychological and cognitive function. The impact of 16PF on the risk of psychological and cognitive skills was the 2nd. Rank 3rd to 6th items were scores for "Cognitive neurological testing", "depression", "Rotate your left hand", "Average when the correct response is made." "Average when the correct response is made" was one of the scoring items for working memory training, "Rotate your left hand" was to assess the women's ability to control their fingers, and "Cognitive neurological testing" was a scale about cognitive neurological testing, which evaluated their cognitive ability from an overall perspective. The red scatter points for "Cognitive Neurological Testing," "Rotate Your Left Hand," and "Average Correct Response" were mostly distributed on the left, while the blue scatter points were on the right. This distribution indicated that the better the female drug users working memory ability, the better their ability to control their fingers, the better their self-evaluated cognitive ability, and the lower the risk of psychological and cognitive function. The mean absolute value of the SHAP of the features was plotted, and it was found that the above 6 variables were important contributing features to explain the psychological and cognitive risk (see Supplementary Figure9B).


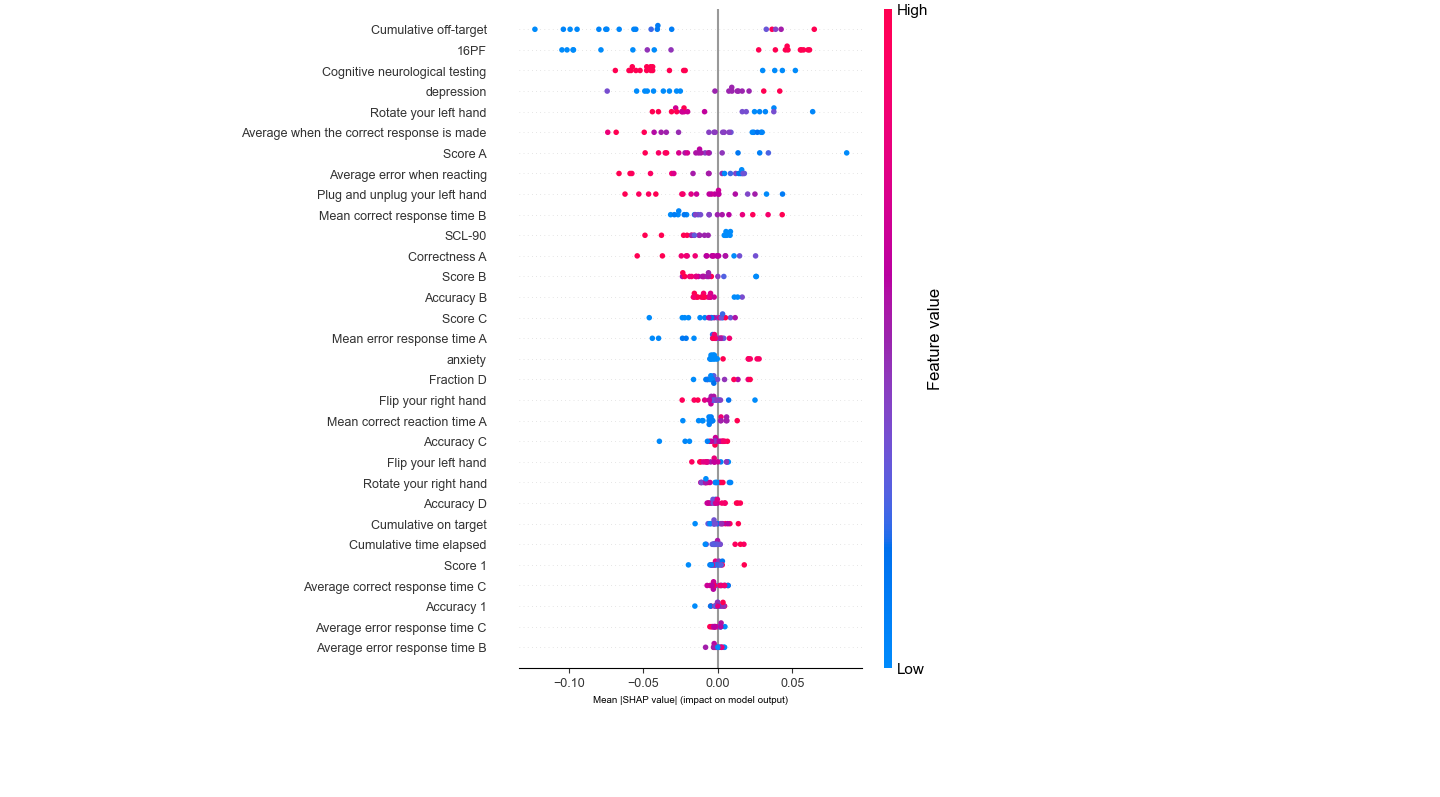


A. Distribution of SHAP values of all samples.


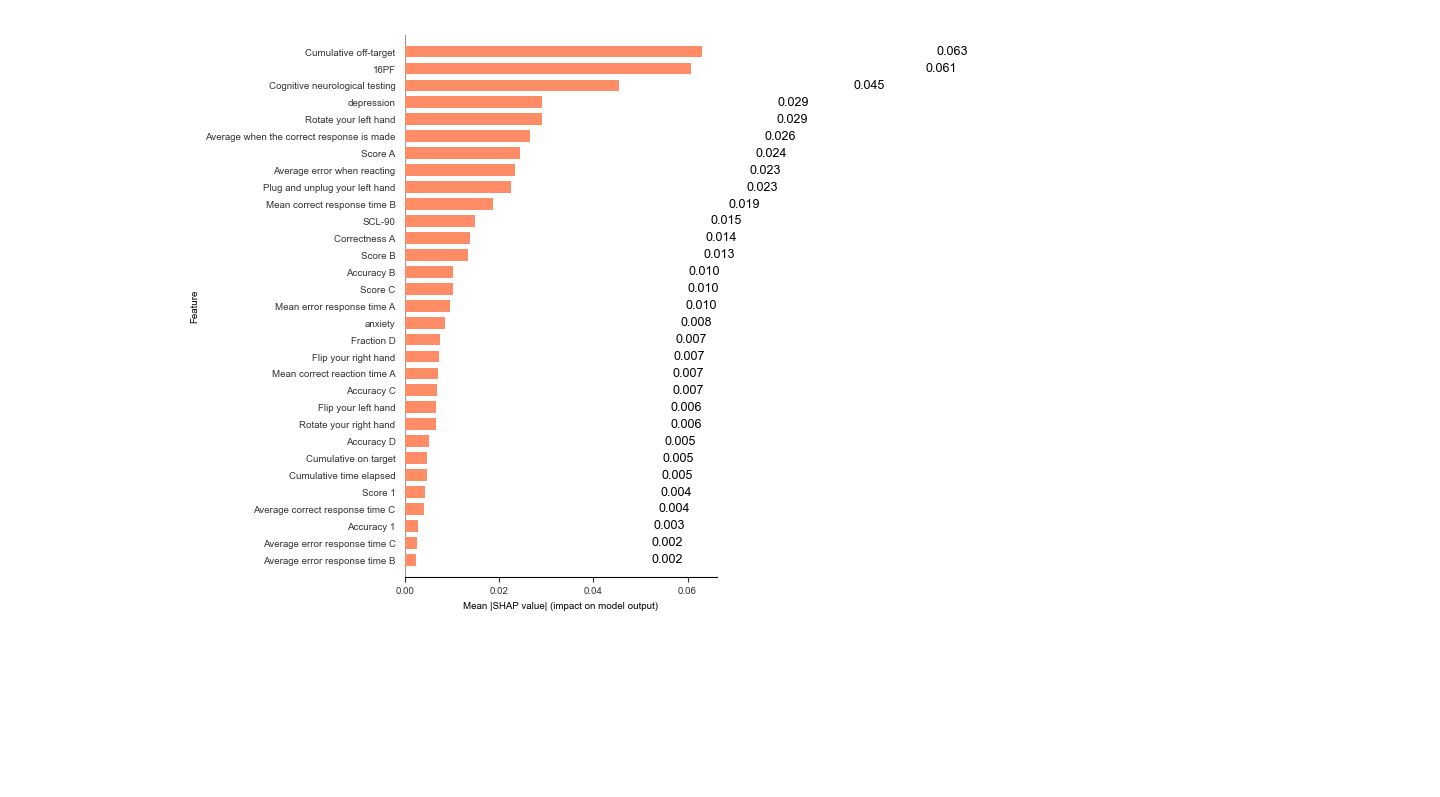


B. Ranking of the absolute value of SHAP value of all features

**Supplementary Figure9**. *SHAP results for the risk of the psychological and cognitive function after oversampling*

## 2.3 The risk of Drug Dependence

**Classification Metrics.** When the risk of drug dependence, the accuracy ranged from 0.56 to 0.78, the total precision ranged from 0.49 to 0.79, the total recall ranged from 0.51 to 0.78, the total F1 score ranged from 0.46 to 0.77, and the AUC value ranged from 0.57 to 0.62(see Table 5). The ROC curves for all classifiers were shown in Supplementary Figure7C. Based on the AUC value as a key indicator, it was found that the LR performed the best. All of LR's indicators ranged from 0.57 to 0.66, and the model performed well.

**Feature Importance.** The distribution of SHAP values corresponding to the 6 variables that predict the risk of drug dependence was shown in the S10A figure. According to the S10A figure, the "Duration of Drug Use" had the greatest impact on the risk of drug dependence, indicating that the longer the "duration of drug use" for female drug users, the greater the probability that the risk of drug dependence might occur. Ranked according to the mean absolute SHAP value of the features (Supplementary Figure10B), the first variable was the "Duration of Drug Use", that was, the "Duration of Drug Use" had the greatest contribution to the risk of drug dependence prediction.

**Supplementary Figure10**. *SHAP results for the risk of the drug dependence after oversampling*


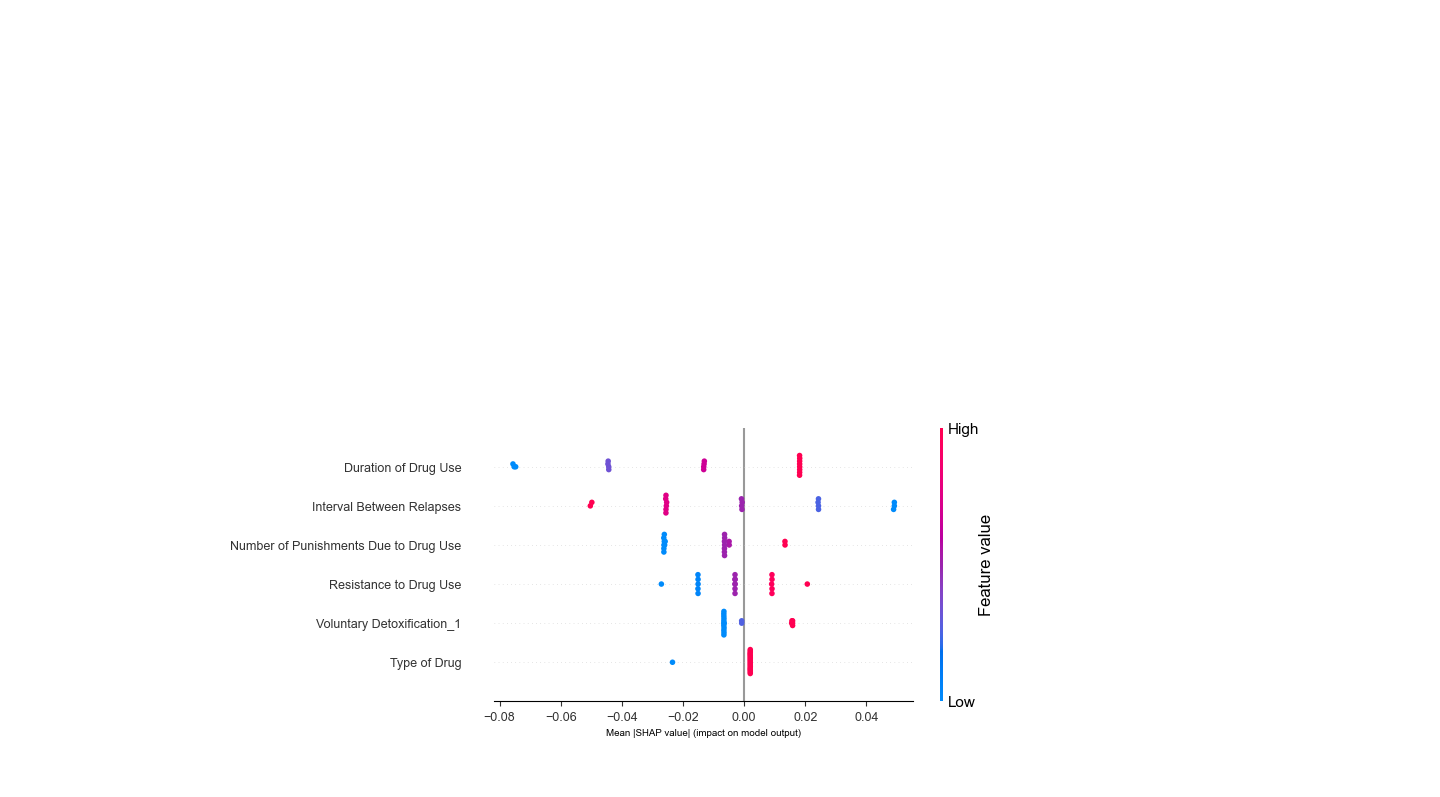


A. Distribution of SHAP values of all samples.


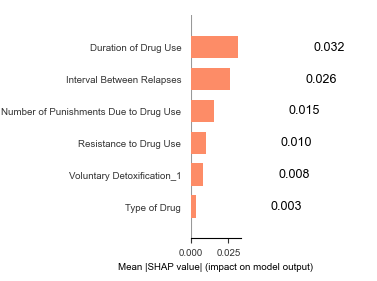


B. Ranking of the absolute value of SHAP value of all features

## 2.4 The Risk of Social Support

**Classification Metrics.** When predicting the risk of social support, the accuracy ranged from 0.46 and 0.59, the total precision ranged from 0.37 and 0.55, the total recall ranged from 0.41 and 0.54, the total F1 score ranged from 0.34 and 0.54, and the AUC value ranged from 0.43 and 0.54(see Table 5). The ROC curves for all classifiers were shown in Supplementary Figure7D. The classifier's performance was selected based on the key indicators of AUC value, and it was found that XGBoost had the best performance. All indicators of XGBoost were between 0.51 and 0.55, and the model indicators were acceptable.

**Feature Importance.** The distribution of SHAP values corresponding to the 18 variables that predict the risk of social support was shown in the S11A figure. According to Supplementary Figure11A, "Social Interaction" had the greatest impact on the risk of social support, indicating that the better the level of "Social Interaction", the more likely the risk of social support is. Ranking 2rd and 3rd were "Vocational Skills Acquired While Incarcerated" and "Fitness Activities Before Incarceration", and the red scatters for these two items were distributed on the left. The red scatter points for these two features were distributed on the left, suggesting that higher levels of "Vocational Skills Acquired While Incarcerated" and "Fitness Activities Before Incarceration" were linked to a lower likelihood of developing social support risk. The 4th indictor that affects the risk of social support is "Educational Background Before Incarceration". The mean SHAP absolute values of the features were plotted, and it was found that the above 4 variables were important contributing features to explain the risk of social support (see Supplementary Figure11B).

**Supplementary Figure11**. *SHAP results for the risk of social support after oversampling*


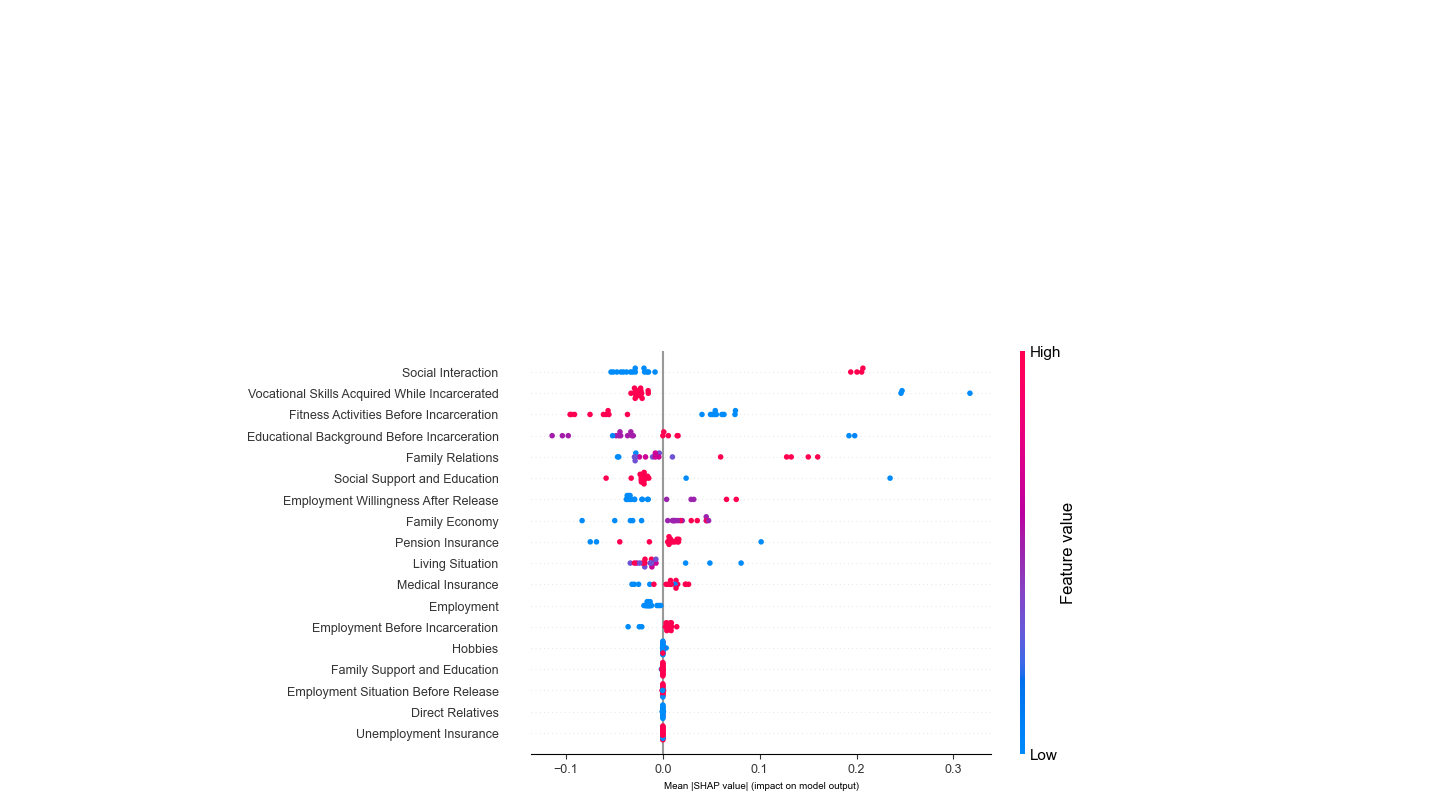


A. Distribution of SHAP values of all samples.


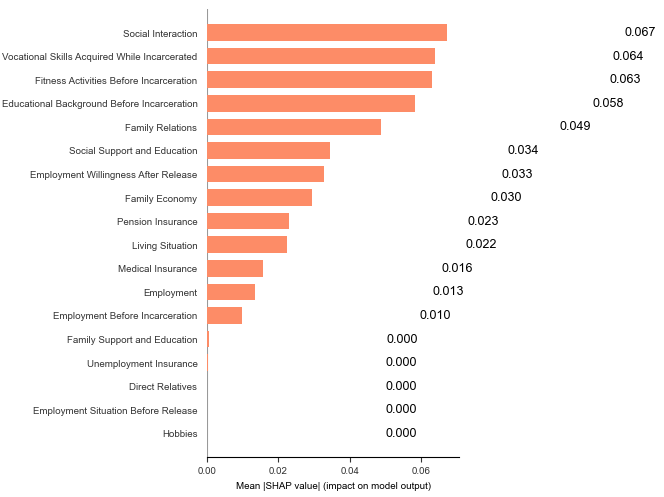


B. Ranking of the absolute value of SHAP value of all features

## 2.5 The Self-Control Risk

**Classification Metrics.** When predicting the self-control risk, the accuracy ranged from 0.74 and 0.83, the total precision ranged from 0.77 and 0.91, the total recall ranged from 0.49 and 0.62, the total F1 score ranged from 0.44 and 0.63, and the AUC value was between 0.53 and 0.68(see Table 5). The ROC curves for all classifiers were shown in Supplementary Figure7E. The performance of the classifier was selected by the key indicators of AUC value, and it was found that the best performer was DT. All indicators of DT were between 0.63 and 0.83, and the model performed well.

**Feature Importance.** The distribution of SHAP values corresponding to the 4 variables that predict the self-control risk was shown in the S12A figure. According to the Supplementary Figure12A, "Compliance and Discipline" had the greatest impact on the self-control risk. Many of the red scatters in "Compliance and Discipline" were on the left and blue on the right, indicating that the higher the number of "Compliance and Discipline", the lower the level of self-control risk. Ranking according to the average absolute SHAP value of the features, the first variable was "Compliance and Discipline", that was, "Compliance and Discipline" had the greatest contribution to the self-control risk prediction (see Supplementary Figure12B).

**Supplementary Figure12**. *SHAP results for the risk of self-control after oversampling*


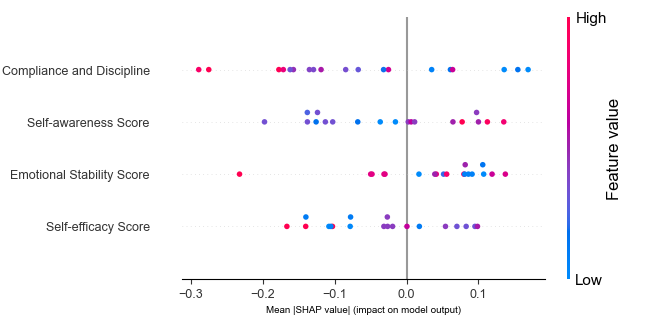


A. Distribution of SHAP values of all samples.


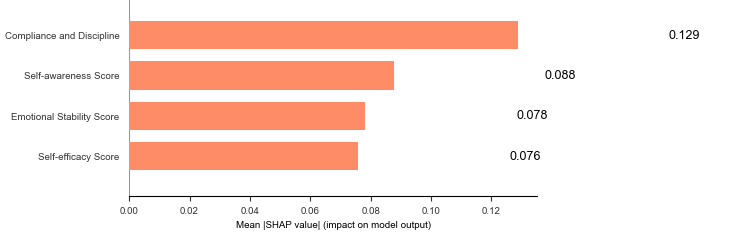


14B. Ranking of the absolute value of SHAP value of all features

# 3 Discussion

## 3.1 Key Features of Physiological Function Risk: Interpretation Based on SHAP

When assessing the risk of physiological function, we included variables of disease, physical fitness and disability, and the classifiers performed well. According to the SHAP analysis results, "chronic diseases" were the most important predictive feature in the classification model based on the original data. After oversampling processing, "physical fitness"—closely related to chronic diseases—became the most influential predictor. Existing studies have indicated that chronic diseases are highly prevalent among individuals hospitalized for substance use[12], and improving physical fitness represents a core objective in exercise interventions for drug users[14]. The physiological risk indicators identified in this study are consistent with the existing literature, further supporting their empirical validity. These findings suggest that in drug rehabilitation practice, the assessment and intervention of physical health among female drug users should prioritize their chronic disease status and physical fitness level.

## 3.2 Key Features of Psychological and Cognitive Function Risk: Interpretation Based on SHAP

When assessing the risk of psychological and cognitive function, we combined mental health test scores (e.g., depression and anxiety test scores), personality test scores, cognitive test scores (e.g., CCAT training scores), and other relevant metrics, and classifiers performed well. SHAP analysis revealed that, based on the original data, the reversed "SCL-90 score", "16PF" factor scores, and four cognitive training items (related to attentional allocation and coordination) were the most important predictors in the model, indicating that more severe psychological symptoms and lower cognitive training performance were associated with higher risk. After oversampling, attention training scores, "16PF" factors, "depression score", working memory training performance, and cognitive assessment scores emerged as the most significant predictors. Among these, cognitive ability and working memory scores contributed negatively to psychological and cognitive risk, suggesting that lower scores in these domains are associated with greater risk. Existing studies have found that depression is common among drug users [8], and females may face an even higher risk of depression, which is consistent with this study’s emphasis on monitoring depressive symptoms in this population. Furthermore, research has reported that cognitive training targeting addiction can help improve executive function and memory in individuals with substance use disorders [1]. This study further highlights the importance of working memory and attention training in addressing psychological and cognitive risk. The 16PF personality factors were also identified as key predictors. Previous studies have suggested that certain personality traits (e.g., perfectionism) may be associated with abstinence outcomes[2]. The current findings not only reinforce the relevance of 16PF in psychological risk assessment but also suggest a potential link with drug dependence behavior. Depression, as a common mental health crisis among drug users [3], may be both a result of substance use[4] and exacerbated by social stigma and discrimination[1]. Therefore, in conducting mental health risk assessment and intervention for female drug users, it is essential to focus comprehensively on cognitive function while also emphasizing identifying and treating mood disorders such as depression to more effectively alleviate their psychosocial burdens.

## 3.3 Key Features of Drug Dependence Risk: Interpretation Based on SHAP

When assessing the risk of drug dependence, the interval between relapses, voluntary detoxification, duration of drug use, resistance to drug use, and type of drug were included with good classifier results. According to SHAP analysis, "duration of drug use" was the key predictor of drug dependence risk, both before and after oversampling. In the original data, its SHAP value was negative, suggesting longer use was linked to lower risk—an unusual pattern that may stem from sample bias. After oversampling, the SHAP value turned positive. This shows longer drug use now corresponds to higher dependence risk, matching clinical knowledge [5-6]. These findings suggest that “duration of drug use” should be regarded as a critical predictive variable in developing risk assessment and intervention strategies for drug dependence among female drug users.

## 3.4 Key Features of Social Support Risk: Interpretation Based on SHAP

When assessing the risk of social support, variables such as basic support (family economy, place of residence, level of education, etc.) and employment were included, and the results showed that these variables were appropriate. According to the SHAP analysis results, before oversampling, "Vocational Skills Acquired While Incarcerated," "Educational Background Before Incarceration," "Unemployment Insurance," and "Employment Situation Before Release" were the most important predictors of social support risk. The negative SHAP values of these factors indicated that higher levels were associated with lower risk. After oversampling, "Social Interaction," "Vocational Skills Acquired While Incarcerated," "Fitness Activities Before Incarceration," and "Educational Background Before Incarceration" emerged as the most significant predictors. Existing research indicates that even when individuals with substance use disorders obtain employment after rehabilitation, such jobs—typically low-paying and unstable—often fail to support long-term recovery or social integration [21]. This aligns with our finding that acquiring practical vocational skills during incarceration is critical to post-release adaptation and stability, underscoring the need for effective training during rehabilitation. Furthermore, lower educational attainment is linked to earlier drug initiation [7], a result consistent with our identification of "Educational Background Before Incarceration" as a key predictor of social support risk. Notably, "Social Interaction" emerged as the primary risk predictor after oversampling. Existing studies indicate that female drug users often need to sever ties with those deemed “detrimental to recovery” [8]. Meanwhile, other research has found that drug users may maintain strong connections with other users to alleviate loneliness [19-20]. This may partly explain the positive contribution of the “social interaction” factor to social support risk. Other factors—including unemployment insurance, physical fitness before incarceration, and pre-release employment—collectively emphasize that deficient social connections and socioeconomic resources heighten social support risk during recovery[8]. These findings suggest that stable economic conditions and healthy interpersonal relationships are crucial components of the social support system for female drug users. Therefore, in building social support systems, in addition to improving educational levels and vocational skills, it is essential to help them identify and avoid detrimental social relationships and guide them in establishing positive and healthy social connections, thereby laying a solid foundation for long-term recovery.

## 3.5 Key Features of Self-Control Risk: Interpretation Based on SHAP

The variables of self-efficacy, self-awareness score, compliance and emotional stability of female drug users were included in self-control risk and the classifiers performed good. According to the SHAP results, "Compliance and Discipline" consistently ranked as the primary risk predictor within its dimension, both before and after oversampling. Before oversampling, higher scores in "Compliance and Discipline" were associated with higher self-control risk, which was inconsistent with clinical observations. However, after oversampling was applied—potentially reducing class imbalance-related bias—lower scores in "Compliance and Discipline" were linked to higher self-control risk, aligning more closely with clinical expectations. Existing research has highlighted the importance of treatment adherence (e.g., participation and cooperation during therapy) among individuals with substance use disorders as a predictor of addiction relapse [9]. Similarly, this study suggests that compliant and disciplined behavior during institutional rehabilitation reflects individuals’ self-control capacity, thereby correlating with self-control risk. These findings imply that when assessing self-control risk in female drug users, attention should be directed toward their daily behavioral performance within institutional settings, particularly their adherence to rules and regulations. Compliance with external norms may serve as a valid indicator of internal self-control levels.

In addition to focusing on the risk of each dimension, we used all the variables of the five dimensions as predictors of the total risk, and the results showed good categorization results in assessing the total risk. Based on the biopsychosocial model of addiction, this study incorporated all 66 indicators as predictors of overall risk in female drug users, with the model demonstrating favorable classification performance. SHAP value analysis revealed that variables from all five dimensions contributed to the total risk, both before and after oversampling. It is worth noting that features such as "Medical Insurance," "Infectious Diseases," and scores from a cognitive training task (one of the EP indicators) exhibited negative SHAP values. Generally, better medical insurance and higher cognitive training performance are associated with lower risk levels, which is consistent with common understanding. However, the negative contribution of the "Infectious Diseases" indicator contradicts intuitive expectations, and this anomalous finding warrants further investigation.

**References**

1. Caetano T, Pinho MS, Ramadas E, Clara C, Areosa T, Dixe M dos A. Cognitive Training Effectiveness on Memory, Executive Functioning, and Processing Speed in Individuals With Substance Use Disorders: A Systematic Review. Frontiers in Psychology. 2021 Aug 13;12. https://doi.org/10.3389/fpsyg.2021.730165

2. Arathil P, Nair A, Narayanan D. Proportion of subjects remaining abstinent following alcohol de-addiction treatment and factors associated with abstinence - A 3 months prospective cohort study. Archives of Mental Health. 2021;22(1):43.‌ https://doi.org/10.4103/amh.amh_49_20

3. Luo D, Tan L, Shen D, Gao Z, Yu L, Lai M, et al. Characteristics of depression, anxiety, impulsivity, and aggression among various types of drug users and factors for developing severe depression: a cross-sectional study. BMC Psychiatry. 2022 Apr 19;22(1).

4. Moustafa AA, Tindle R, Cashel S, Parkes D, Mohamed E, Abo Hamza E. Bidirectional relationship between heroin addiction and depression: Behavioural and neural studies. Current Psychology. 2020 Aug 31;41.‌ https://doi.org/10.1007/s12144-020-01032-4

5. Moeeni M, Razaghi EM, Ponnet K, Torabi F, Shafiee SA, Pashaei T. Predictors of time to relapse in amphetamine-type substance users in the matrix treatment program in Iran: a Cox proportional hazard model application. BMC Psychiatry. 2016 Jul 26;16(1).‌ https://doi.org/10.1186/s12888-016-0973-8

6. Blum K, Chen AL, Giordano J, Borsten J, Chen TJ, Hauser M, et al. The Addictive Brain: All Roads Lead to Dopamine. Journal of Psychoactive Drugs. 2012 Apr;44(2):134–43.‌ https://doi.org/10.1080/02791072.2012.685407

7. Shaw., A. Women in mid-life and older age in recovery from illicit drug use: connecting and belonging. Frontiers in Psychiatry,2023, 14, 1221500. https://doi.org/10.3389/fpsyt.2023.12215

8. Favril L. Drug Use before and during imprisonment: Drivers of Continuation. International Journal of Drug Policy [Internet]. 2023 May 1;115(1):104027. Available from: https://www.sciencedirect.com/science/article/pii/S0955395923000762

9. Peng W, Zhang H, Yang J, Wang J, Kang J, Zhu R, et al. Group cognitive behavioral therapy as an effective approach for patients with alcohol dependence: A perspective study. Medicine. 2022 Sep 9;101(36):e30459.‌https://doi.org/10.1097/MD.0000000000030459
